# 7*α*-Hydroxypaxilline, a Natural Product Isolated from Mangrove Endophytic Fungi, Inhibits Protein Translation in *Vibrio alginolyticus*

**DOI:** 10.3390/md24100334

**Published:** 2026-09-25

**Authors:** Pei Lv, Xiaobo Liu, Xinlan Xing, Guaijie Qiao, Senqian Li, Yuying Xie, Hongming Cao, Yuyue Qin

**Affiliations:** Pinglu Canal and Beibu Gulf Coastal Ecosystem Observation and Research Station of Guangxi, Guangxi Key Laboratory of Marine Environmental Disaster Processes and Ecological Protection Technology, College of Marine Sciences, Beibu Gulf University, Qinzhou 535011, China; 18928471091@163.com (P.L.); chemweaver@163.com (X.L.); l2909909@163.com (X.X.); 18977215324@163.com (G.Q.); qnmd6679@163.com (S.L.); 19152632615@163.com (Y.X.)

**Keywords:** mangrove-derived fungus, natural product, *Vibrio alginolyticus*, antibacterial activity

## Abstract

*Vibrio alginolyticus*, a prevalent marine Gram-negative pathogenic bacterium, can cause vibriosis in aquaculture and pose potential threats to human health. The excessive abuse of antibiotics has aggravated the multidrug resistance of bacteria, driving researchers to explore natural antibacterial substances from mangrove endophytic fungi. In this study, four known indole-diterpenoids, 7*α*-hydroxypaxilline (**1**), paspaline (**2**), paspalinine (**3**), 4a-demethylpaspaline-4a-carboxylic acid (**4**), were isolated the marine-derived fungus *Penicillium janthinellum* D131. Among them, compound **1** exhibited superior inhibitory activity against *Vibrio alginolyticus*, with a minimum inhibitory concentration (MIC) of 0.781 μg/mL, which was superior to chloramphenicol (1.56 μg/mL). A series of phenotypic experiments confirmed the potent bactericidal activity of this compound. The results of multi-omics analysis and Western blot assays demonstrated that the compound disrupts the 50S ribosome-mediated translation process in bacteria. Molecular docking analysis and kinetic simulation were further performed to identify the target site, and the results revealed that the compound interacts with 23S ribosomal RNA (23S rRNA). In vitro translation assays further verified that the compound blocks bacterial protein synthesis by targeting 23S rRNA. This study reports the excellent anti-vibrio activity of **1**, which is superior to chloramphenicol, and clarifies its ribosome-targeting mechanism of action. These findings provide a theoretical basis for the research and development of novel antibacterial drugs for aquaculture and broaden the potential applications of lead compounds.

## 1. Introduction

*V. alginolyticus*, a salt-tolerant Gram-negative pathogen widespread in coastal aquaculture [1], causes severe vibriosis in shrimp [2,3], fish, and corals and also triggers human wound infections and gastroenteritis. Antibiotic overuse has fostered its multidrug resistance, creating an urgent need for low-toxic natural antimicrobials [4].

Mangrove endophytic fungi, as the second largest ecological group of marine-derived fungi, have developed various metabolic pathways and adaptation mechanisms in the unique marine environment, thereby yielding secondary metabolites notable for both structural novelty and potent bioactivity [5]. However, few studies have systematically explored mangrove fungal metabolites against resistant *V. alginolyticus* and elucidated their molecular mechanisms, representing an important research gap [6].

Indole diterpenoids are an important class of secondary metabolites produced by marine-derived fungi, featuring a common core structure comprising an indole derived from indole-3-glycerol phosphate, and a cyclic diterpene skeleton derived from geranylgeranyl diphosphate [7,8]. Indole diterpenoids have attracted extensive attention for their diverse skeletons [9]. Among the known indole diterpenoids, paspaline-type and paxiline-type congeners are the largest classes with the most multitudinous structures [10]. Owing to unique and complex structures with multiple chiral centers, indole diterpenoids reveal a wide range of bioactivities, encompassing anti-insectan activity [11], cytotoxicity [8,9], tremorgenic activity [12], antibacterial activities [9], cholesterol acyltransferase (ACAT) inhibitors [13], tremorgenic and neurotoxic activities [14,15], M phase cell cycle inhibitors [16], and in vitro anti-proliferative, anti-insectan activities [17], etc., which have attracted considerable attention from chemists and pharmacologists.

In this study, four known indole-diterpenoids, 7*α*-hydroxypaxilline (**1**) [18], paspaline (**2**) [19,20], paspalinine (**3**) [21], and 4a-demethylpaspaline-4a-carboxylic acid (**4**), were isolated and identified from the rice solid-state fermentation of *P. janthinellum* D131. Comprehensive antibacterial screening and multilevel mechanistic research integrating cytology, multi-omics, molecular docking, and md simulations are then performed. This work aims to promote the development and utilization of mangrove endophytic fungi; clarify the molecular mechanism underlying the anti-vibrio pathway of their bioactive components; provide a theoretical basis for the development of eco-friendly antibacterial agents for the prevention and control vibriosis; and help mitigate bacterial resistance in aquaculture, thereby expanding the potential applications of these lead compounds in eco-friendly antibacterial research for aquaculture.

## 2. Results

### 2.1. Identification of the Compound Structures

Based on ^1^H NMR and ^13^C NMR data, HR-ESI-MS, and by comparison with the data previously reported in the literature, compounds **1**–**4** were determined to be 7*α*-hydroxypaxilline (**1**) [18], paspaline (**2**) [19,20], paspalinine (**3**) [21], and 4a-demethylpaspaline-4a-carboxylic acid (**4**) (Figure 1).

Since the ^13^C NMR data of 7*α*-hydroxypaxilline (**1**) [18] have not been previously published, the ^13^C NMR data of 7*α*-hydroxypaxilline (**1**) are reported here for the first time (Supplementary Materials, Table S1). The structure and absolute configuration of 7*α*-hydroxypaxilline (**1**) were elucidated by HR-ESI-MS and 1D and 2D NMR spectroscopic data, together with quantum chemical electronic circular dichroism (ECD) calculation.

Compound **1**: white solid powder, Its molecular formula was established to be C_27_H_33_NO_5_ with 12 degrees of unsaturation on the basis of HR-ESI-MS ion at *m*/*z* 452.2431 [M + H]^+^ (calcd 452.2437). By detailed comparison of the ^1^H NMR data of **1** with those of 7*α*-hydroxypaxilline, [18] indicated that **1** has the same planar structure as 7*α*-hydroxypaxilline. This deduction was determined by the ^1^H−^1^H COSY correlations between H_2_-5/H_2_-6, H_2_-14/H_2_-15, H_2_-15/H-16, H-16/H_2_-17, H-20/H-21, H-21/H-22, and H-22/H-23, and the HMBC correlations from NH-1 to C-2/C-18/C-19/C-23, H_2_-5 to C-7/C-13, H_2_-6 to C-4/C-7, H-9 to C-7/C-10, H-11 to C-7/C-12/C-13, H_2_-14 to C-13, H_2_-15 to C-3/C-13, H_2_-17 to C-2/C-3/C-18, H-20 to C-22/C-24, H-21 to C-19, H-22 to C-24, H-23 to C-19/C-21, H_3_-25 to C-2/C-4/C-16, H_3_-26 to C-3/C-5/C-13, H_3_-28 to C-9/C-27, H_3_-29 to C-9/C-27/C-28, OH-13 to C-17, and OH-27 to C-27/C-28/C-29 (Supplementary Materials, Figure S1A).

The relative configuration of **1** was elucidated by the NOESY correlations (Supplementary Materials, Figure S1B). The cross-peaks of H-16/H_3_-26 assigned these protons as *β*-oriented, whereas the NOESY correlations of H_3_-25/OH-13 and OH-13/H-9 assigned H-9, H_3_-25, and OH-13 as *α*-oriented. This is consistent with the NOESY correlations of 7*α*-hydroxypaxilline [18] reported in the literature. Because of lacking diagnostic NOESY correlations, the relative configurations of C-7 could not be directly assigned. To further elucidate the relative configuration of **1**, the ^13^C NMR theoretical calculations of two possible diastereomers, (7S*)-**1A** and (7R*)-**1B,** were performed at the MPW1PW91/6-311+G(d,p) level of theory [22]. The highly matched linear dependence of (7S*)-**1A** (*R*^2^ = 0.9986) between the experimental and calculated ^13^C NMR chemical shifts (Supplementary Materials, Figure S2) with a DP4+ probability of 100% (Supplementary Materials, Figure S3) [22] further confirmed that the relative configuration of **1** was established as 3S*, 4R*, 7S*, 9R*, 13S*, 16S*. Finally, the calculated ECD spectrum of (3*S*, 4*R*, 7*S*, 9*R*, 13*S*, 16*S*)-**1a** showed good agreement with the experimental spectrum of **1** (Supplementary Materials, Figure S1C). Therefore, the absolute configuration of **1** was assigned as 3*S*, 4*R*, 7*S*, 9*R*, 13*S*, 16*S *(Figure 1).

7*α*-Hydroxypaxilline (**1**): white solid powder, [*α* ${]}_{D}^{25}$ −15.7 (*c* 0.10, DMSO), HR-ESI-MS *m*/*z* 452.2431 [M + H]^+^ (calcd 452.2437). ^1^H NMR (600 MHz, DMSO-*d*_6_) *δ*_H_: 10.76 (s, 1H, NH-1), 7.27 (t, *J* = 7.0 Hz, 2H, H-20/H-23), 6.94 (t, *J* = 7.6 Hz, 1H, H-22), 6.89 (d, *J* = 7.0 Hz, 1H, H-21), 5.76 (s, 1H, H-11), 5.03 (s, 1H, OH-7), 4.32 (s, 1H, OH-27), 4.16 (s, 1H, H-9), 2.77 (m, 1H, H-16), 2.62 (m, 1H, H-17a), 2.51 (m, 1H, H-5a), 2.33 (m, 1H, H-17b), 2.18 (m, 1H, H-6a), 2.13 (m, 1H, H-6b), 1.94 (m, 1H, H-15a), 1.85 (m, 1H, H-14a), 1.83 (m, 1H, H-5b), 1.70 (m, 1H, H-14b), 1.64 (m, 1H, H-15b), 1.26 (s, 3H, H-25), 1.19 (s, 3H, H-28), 1.18 (s, 3H, H-29), 0.93 (s, 3H, H-26). ^13^C NMR (150 MHz, DMSO-*d*_6_) *δ*_C_: 198.26 (C-10), 161.43 (C-12), 152.68 (C-2), 139.79 (C-24), 124.49 (C-19), 120.47 (C-11), 119.31 (C-22), 118.47 (C-21), 117.70 (C-20), 114.97 (C-18), 111.81 (C-23), 93.76 (C-7), 78.06 (C-9), 76.09 (C-13), 71.14 (C-27), 50.35 (C-3), 49.40 (C-16), 42.56 (C-4), 35.07 (C-6), 32.57 (C-14), 26.90 (C-17), 26.24 (C-29), 26.09 (C-28), 25.28 (C-5), 20.96 (C-15), 18.99 (C-26), 16.20 (C-25) (Supplementary Materials, Figures S4 and S5, Table S1).

Paspaline (**2**): white solid powder, HR-ESI-MS *m*/*z* 422.3057 [M + H]^+^ (calcd 422.3059). ^1^H NMR (600 MHz, DMSO-*d*_6_) *δ*_H_: 10.58 (s, 1H, NH-1), 7.26 (d, *J* = 3.6 Hz, 1H, H-23), 7.25 (d, *J* = 3.2 Hz, 1H, H-20), 6.93 (t, *J* = 7.3 Hz, 1H, H-22), 6.89 (t, *J* = 7.3 Hz, 1H, H-21), 4.10 (s, 1H, OH-28), 3.17 (m, 1H, H-9), 3.09 (dd, *J* = 11.6, 2.6 Hz, 1H, H-7), 2.97 (dd, *J* = 11.6, 3.5 Hz, 1H, H-16), 2.65 (m, 1H, H-17a), 2.56 (m, 1H, H-11a), 2.24 (m, 1H, H-17b), 1.93 (d, *J* = 12.6 Hz, 1H, H-5a), 1.79 (m, 1H, H-6a), 1.73 (m, 1H, H-5b), 1.70 (m, 1H, H-11b), 1.63 (m, 1H, H-14a), 1.57 (m, 1H, H-15a), 1.55 (m, 1H, H-10a), 1.53 (m, 1H, H-6b), 1.51 (m, 1H, H-10b), 1.47 (m, 1H, H-13), 1.45 (m, 1H, H-14b), 1.32 (m, 1H, H-15b), 1.10 (s, 3H, H-27), 1.04 (s, 6H, H-29/H-30), 0.96 (s, 3H, H-25), 0.82 (s, 3H, H-26). ^13^C NMR (150 MHz, DMSO-*d*_6_) *δ*_C_: 151.17 (C-2), 140.25 (C-24), 124.44 (C-19), 119.31 (C-22), 118.39 (C-21), 117.58 (C-20), 115.82 (C-18), 111.88 (C-23), 85.24 (C-7), 84.60 (C-9), 70.40 (C-28), 52.47 (C-3), 48.57 (C-16), 45.89 (C-13), 40.06 (C-4), 37.24 (C-11), 36.08 (C-12), 32.46 (C-5), 27.10 (C-17), 26.95 (C-30), 25.09 (C-6), 24.63 (C-29), 24.54 (C-15), 21.53 (C-14), 21.08 (C-10), 19.44 (C-26), 14.57 (C-25), 12.64 (C-27) (Supplementary Materials, Figures S12 and S13, Table S2).

Paspalinine (**3**): white solid powder, HR-ESI-MS *m*/*z* 434.2330 [M + H]^+^ (calcd 434.2331). ^1^H NMR (600 MHz, DMSO-*d*_6_) *δ*_H_: 10.60 (s, 1H, NH-1), 7.28 (m, 1H, H-20), 7.26 (m, 1H, H-23), 6.93 (t, *J* = 7.5 Hz, 1H, H-22), 6.90 (t, *J* = 7.2 Hz, 1H, H-21), 5.73 (s, 1H, H-11), 5.08 (s, 1H, OH-13), 4.41 (s, 1H, H-9), 2.73 (m, 1H, H-16), 2.69 (m, 1H, H-17a), 2.60 (m, 1H, H-5a), 2.45 (m, 1H, H-6a), 2.32 (m, 1H, H-17b), 2.01 (m, 1H, H-6b), 1.95 (m, 1H, H-15a), 1.91 (m, 1H, H-14a), 1.86 (m, 1H, H-5b), 1.80 (m, 1H, H-14b), 1.63 (m, 1H, H-15b), 1.36 (s, 3H, H-25), 1.31 (s, 3H, H-28), 1.11 (s, 3H, H-29), 1.08 (s, 3H, H-26). ^13^C NMR (150 MHz, DMSO-*d*_6_) *δ*_C_: 196.95 (C-10), 169.71 (C-12), 152.82 (C-2), 140.10 (C-24), 124.64 (C-19), 119.27 (C-11), 118.46 (C-21), 117.69 (C-22), 116.80 (C-20), 114.87 (C-18), 111.90 (C-23), 104.21 (C-7), 86.89 (C-9), 77.92 (C-27), 75.95 (C-13), 50.96 (C-3), 48.47 (C-16), 39.15 (C-4), 31.83 (C-14), 28.54 (C-28), 27.99 (C-6), 27.20 (C-17), 25.94 (C-5), 22.92 (C-29), 22.52 (C-26), 20.96 (C-15), 16.38 (C-25) (Supplementary Materials, Figures S15 and S16, Table S2).

4a-Demethylpaspaline-4a-carboxylic acid (**4**): white solid powder, HR-ESI-MS *m*/*z* 450.2643 [M − H]^−^ (calcd 450.2644). ^1^H NMR (600 MHz, DMSO-*d*_6_) *δ*_H_: 10.60 (s, 1H, NH-1), 7.26 (m, 1H, H-23), 7.25 (m, 1H, H-20), 6.92 (t, *J* = 7.6 Hz, 1H, H-22), 6.89 (t, *J* = 7.6 Hz, 1H, H-21), 4.06 (s, 1H, OH-28), 3.12 (m, 1H, H-9), 3.10 (m, 1H, H-7), 2.62 (m, 1H, H-16), 2.57 (m, 1H, H-17a), 2.54 (m, 1H, H-6a), 2.36 (m, 1H, H-11a), 2.24 (m, 1H, H-17b), 1.98 (m, 1H, H-5a), 1.84 (m, 1H, H-5b), 1.80 (m, 1H, H-14a), 1.70 (m, 1H, H-15a), 1.67 (m, 1H, H-13), 1.64 (m, 1H, H-10a), 1.62 (d, *J* = 7.2 Hz, 1H, H-14b), 1.51 (m, 1H, H-15b), 1.27 (m, 1H, H-6b), 1.23 (m, 1H, H-11b), 1.19 (m, 1H, H-10b), 1.09 (s, 1H, H-30), 1.00 (s, 1H, H-29), 0.97 (s, 1H, H-25), 0.92 (s, 1H, H-26). ^13^C NMR (150 MHz, DMSO-*d*_6_) *δ*_C_: 175.29 (C-27), 150.96 (C-2), 140.24 (C-24), 124.40 (C-19), 119.36 (C-22), 118.42 (C-21), 117.62 (C-20), 115.88 (C-18), 111.90 (C-23), 84.24 (C-9), 83.61 (C-7), 70.35 (C-28), 51.69 (C-3), 48.63 (C-16), 47.26 (C-12), 46.03 (C-13), 40.06 (C-4), 35.28 (C-11), 32.41 (C-5), 27.03 (C-17/C-30), 25.18 (C-6), 24.77 (C-15), 24.44 (C-14), 23.41 (C-29), 22.81 (C-10), 16.17 (C-26), 14.51 (C-25) (Supplementary Materials, Figures S18 and S19, Table S2).

**Figure 1:**
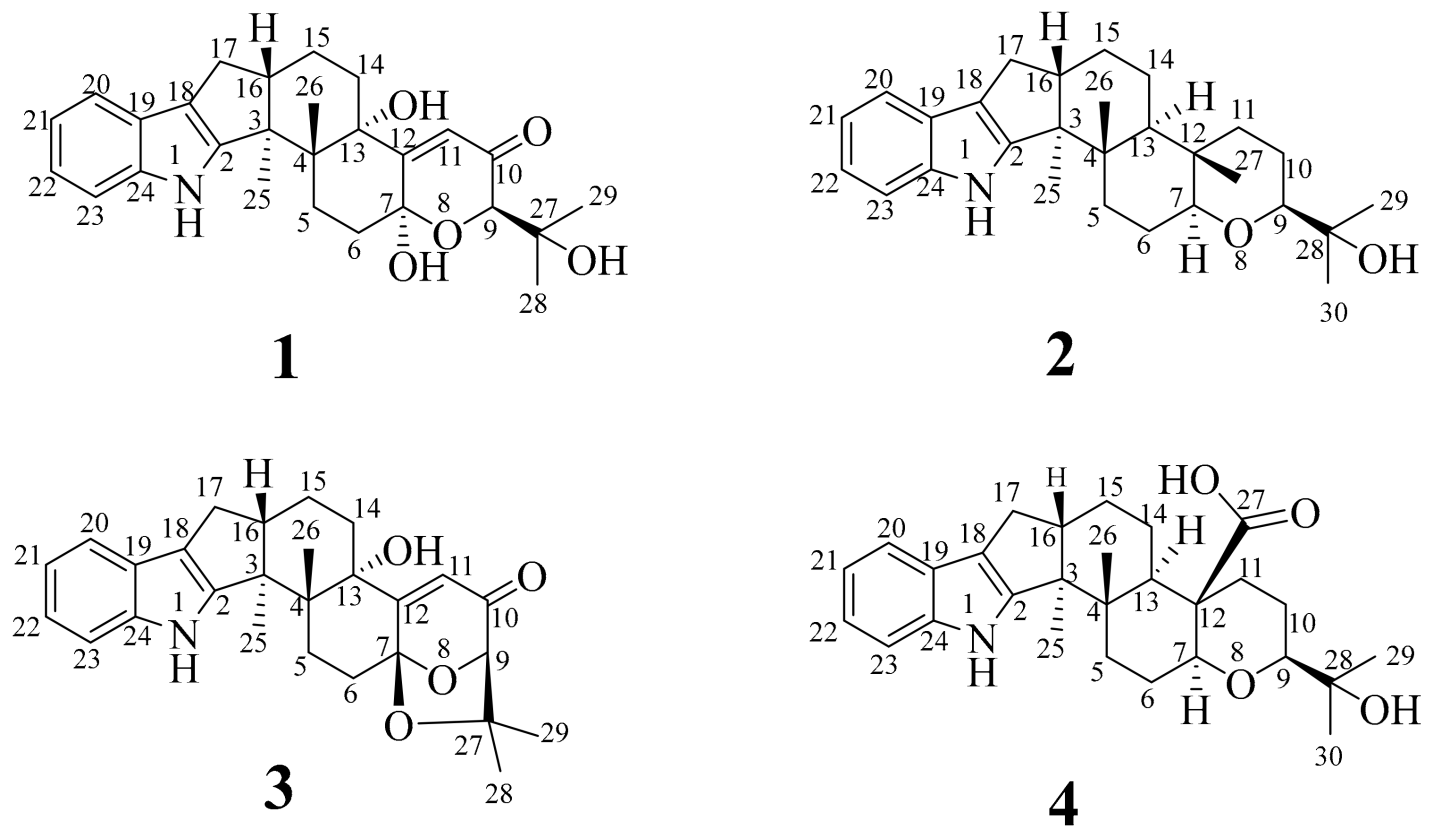
The chemical structures of compounds **1**–**4**.

### 2.2. Antibacterial Activity of Compounds Against V. alginolyticus

The in vitro antibacterial activities of compounds **1**–**4** against *V. alginolyticus* were determined via the two-fold broth microdilution method in 96-well plates. The results revealed that only compound **1** exerted potent inhibitory activity against the strain, with an MIC value of 0.781 μg/mL. The MIC of chloramphenicol (positive control) was 1.56 μg/mL, demonstrating that compound **1** possessed stronger antibacterial activity than chloramphenicol.

### 2.3. The Effect of ***1*** on V. alginolyticus Growth Curves

With 1% DMSO as solvent control, *V. alginolyticus* growth curves were monitored via OD_600_. The 1/8 × MIC group showed slightly suppressed growth, while 1 × MIC and 2 × MIC fully blocked bacterial proliferation. Compound **1** exhibited concentration-dependent bactericidal effects, possessing great potential as a natural antibacterial candidate (Figure 2A).

### 2.4. Leakage of DNA and Proteins

Damaged bacterial membranes cause cytoplasmic DNA and protein leakage. After treating *V. alginolyticus* with gradient concentrations of **1**, extracellular DNA (Figure 2B) and protein contents (Figure 2C) increased significantly in a dose-dependent manner versus the control. Compound **1** impairs membrane integrity and triggers intracellular substance leakage, ultimately killing bacteria. Hence, its antibacterial activity is efficacious, as evidenced by the disruption of the bacterial cell membrane.

### 2.5. Intracellular ROS Production

Excessive intracellular ROS is another antibacterial indicator measured by DCFH-DA staining. Compared with the control, compound **1** drastically raised ROS levels in *V. alginolyticus* (*p* < 0.001) in a dose-dependent manner from 0.78 (1 × MIC) to 3.12 (4 × MIC) μg/mL (Figure 2D). ROS accumulation partially accounts for its bactericidal effect.

### 2.6. Plasma Stability Assays of Compound **1**

As plasma stability is a critical determinant for efficacy of a compound in vivo, we first evaluated the stability of **1** under physiological conditions prior to the in vivo antibacterial experiment. As shown in Figure 3A, the hemolysis assay demonstrated that **1** induced no obvious hemolysis at concentrations up to 50 μg/mL, which confirmed its good biocompatibility with blood constituents. These findings imply that **1** exhibits favorable plasma stability.

### 2.7. Inhibition Biofilm Formation

Since bacterial membrane damage is an effective strategy against biofilm maturation and **1** was verified to destroy the membrane of *V. alginolyticus*, we next evaluated its anti-biofilm potential. After culturing bacteria with subinhibitory doses (allowing viable growth), biofilm formation was assessed over 24 h via crystal violet staining. The results demonstrated that **1** significantly reduced biofilm formation (Figure 3B).

### 2.8. Fluorescence Microscopy (FM)

Building upon the SEM observations, DAPI (4′,6-diamidino-2-phenylindole)/PI (propidium iodide) double staining was further adopted to evaluate the membrane integrity of *V. alginolyticus* after incubation with **1**. DAPI permeates intact membranes and stains nuclear DNA, emitting blue fluorescence, whereas PI enters only cells with compromised membranes and stains DNA red. As shown in Figure 4A, the control group displayed bright blue fluorescence with minimal red signal, consistent with intact plasma membranes and viable bacteria. However, markedly enhanced red PI fluorescence was detected in the compound-treated group, proving that **1** destroys bacterial membrane integrity and ultimately triggers bacterial death.

### 2.9. Scanning Electron Microscopy (SEM)

To investigate the potential effect of **1**, SEM was employed to examine morphological changes in *V. alginolyticus* cells. As shown in Figure 4B, untreated control cells displayed intact, smooth surfaces with typical rod-shaped morphology. In contrast, treatment with **1** suffered severe membrane damage, featuring obvious surface rupture, shrinkage and destroyed structural integrity. These observations indicate that **1** compromises the cell membrane of *V. alginolyticus*.

### 2.10. Transcriptome Analysis of Compound **1**-Treated V. alginolyticus

To explore the antibacterial targets of **1**, transcriptome sequencing was performed on *V. alginolyticus* treated with compound **1** at 1 × MIC (0.78 μg/mL). In total, 52.68 Gb of clean data were generated from six samples. Each sample yielded over 2.95 Gb clean data with Q30 values above 97.21%. Based on the reference genome GCF_050507685.1, 4340 expressed genes were annotated, including 4236 known genes, 72 novel genes, and 32 sRNAs. With thresholds of |log_2_FC| ≥ 1 and padjust < 0.05, DESeq2 analysis identified 1441 differentially expressed genes (DEGs), consisting of 692 upregulated and 749 downregulated genes. GO and KEGG enrichment analyses revealed that transmembrane transporter activity and transporter activity were the most enriched GO terms (Figure 5A), while the ribosome pathway showed the highest KEGG enrichment (Figure 5B). These results indicated that **1** mainly interferes with transmembrane transport and protein synthesis, which are presumed to be its potential action targets in *V. alginolyticus*.

### 2.11. PCR (qRT-PCR) Analysis

Transcriptome sequencing data and gene quantification results calculated by the 2^−ΔΔCt^ method from qRT-PCR revealed that all seven selected genes exhibited significantly differential expression upon compound-**1** treatment. These seven genes (*punC*, *ACQY1V_RS16500*, *rplM*, *rplX*, *rplE*, *rplU*, *rplQ*) were markedly upregulated (Figure 5C). Among the 2^−ΔΔCt^ quantitative comparisons of qPCR, *rplE* displayed the most prominent expression change. Five of the seven genes encode proteins of the 50S ribosomal subunit. Collectively, *V. alginolyticus* 50S ribosomal subunit is highly susceptible to **1**, and the compound’s inhibitory effect on the 50S subunit is largely mediated via regulation of *rplE*.

### 2.12. Label-Free Quantitative Proteomics Analysis of Compound** 1**

Proteomic analysis was conducted to uncover the antibacterial mechanism of **1** against *V. alginolyticus*. GO annotation revealed the most differential proteins belonged to cellular anatomical structure (CC); KEGG annotation concentrated differential proteins in metabolic overview pathways, and EggNOG classification highlighted amino acid transport/metabolism and transcription. GO enrichment showed no prominent pathways (Figure 6A,B), while KEGG enrichment identified phenylalanine, tyrosine, and tryptophan biosynthesis as the top enriched pathway (Figure 6C,D). Compound **1** severely disturbs bacterial amino acid metabolism, causing disruptions to cellular structure and core physiological processes.

### 2.13. Target Protein Expression

Combined with transcriptomic, qRT-PCR, and proteomic data analysis, compound **1** is speculated to target the 50S ribosomal subunit of *V. alginolyticus* and block protein translation. Notably, the RplE protein screened via transcriptome and qRT-PCR was not among the most significantly enriched proteins in proteomics. Western blot assays were therefore conducted for further verification. Comparison among the 1/4 × MIC, 1/2 × MIC treatment groups, and the blank control group revealed that the overall band gray values of treatment groups were higher than those of the control, and the increase in gray values exhibited a dose-dependent trend (Figure 7). These findings confirmed that *rplE* was significantly upregulated in *V. alginolyticus* under subinhibitory concentrations of **1**.

### 2.14. Docking Analysis

Compound **1** binds to Domain II of the 50S 23S rRNA, forming hydrogen bonds with G949 (3.03 Å), C965 (2.91 Å), and C964 (2.80 Å), as revealed by AutoDock 1.5.6. These residues all lie within Domain II, which in the three-dimensional fold projects toward the peptidyl transferase center (PTC) and also participates in A/P-site tRNA binding. RplE (L5) controls elongation efficiency and 50S maturation. Compound **1** disturbs 23S rRNA folding and reduces functional 50S production. *V. alginolyticus* activates ribosome biogenesis compensation and markedly upregulates *rplE*. AutoDock Vina gives a binding affinity of −11.0 kcal/mol, suggesting stable binding between **1** and 23S rRNA (Figure 8).

### 2.15. MD Simulation Analysis

Molecular dynamics (MD) simulations were performed to reveal the antibacterial mechanism of compound **1** by investigating its interactions with 23S rRNA, with initial structural coordinates obtained from molecular docking results. The radius of gyration (Rg) and its decomposition along the three principal axes (RgX, RgY, RgZ) were calculated to evaluate the effect of ligand binding on the overall RNA conformation. The overall Rg exhibited minimal fluctuations and remained stable at approximately 8.0 nm throughout the 100 ns simulation, and the axial components RgX, RgY, and RgZ followed a consistent trend with similarly small oscillations. Notably, RgX and RgZ displayed repeated minor cycles of compaction and separation over the trajectory. These results indicate that the binding site itself did not undergo large-scale movements upon compound **1** binding to 23S rRNA; instead, only subtle, repeated compaction–relaxation motions occurred along the RgX and RgZ directions (Figure 9A). Hydrogen bond analysis revealed that a relatively large number of hydrogen bonds were present in the initial state of the ligand–23S rRNA complex, consistent with the molecular docking results. Over the 100 ns simulation, however, the hydrogen bond count was high only at the initial stage and subsequently stabilized at approximately 1–2, with an average of only one persistent hydrogen bond (Figure 9B). This finding suggests that the tight binding of compound **1** to 23S rRNA is not primarily driven by hydrogen bonds; rather, hydrogen bonds serve mainly to anchor the ligand at the target residue positions. Solvent-accessible surface area (SASA), which measures the portion of the system surface accessible to water molecules, decreased from 4700 nm^2^ to 4400 nm^2^ during the first 60 ns of the simulation and thereafter remained stable. This reduction indicates the formation of a compact binding interface and the burial of hydrophobic surfaces upon ligand binding. The 300 nm^2^ decrease in SASA further supports a hydrophobic-driven binding mode, whereby the 23S rRNA–compound 1 complex adopts a more compact conformation (Figure 9C). Root-mean-square fluctuation (RMSF) analysis was performed to assess the residue-level flexibility of each nucleotide in 23S rRNA, with particular focus on the molecular docking binding-site region (G949/C964/C965). Within the residue interval 949–969, the RMSF values ranged from approximately 0.3 to 0.7 nm, representing a medium-to-low level relative to the overall structure and indicating that the binding region itself is relatively stable, permitting precise ligand binding. However, this interval was immediately flanked by the highest RMSF peaks on both sides, suggesting that the ligand-binding region is sandwiched between two highly flexible regions. Combined with the SASA-derived compaction and the hydrophobic-driven binding evidence, these results indicate that after positional binding to 23S rRNA, compound **1** is compressed by the surrounding flexible regions, and hydrophobic stacking interactions drive the conformation to become more compact (Figure 9D). Gibbs free energy landscape (FEL) analysis, constructed based on principal component analysis (PCA), revealed a single deep minimum (ΔG = 0 kJ/mol) at RMSD ≈ 1.9 nm and Rg ≈ 8.15 nm during the 100 ns simulation, with an energy barrier of approximately 14 kJ/mol separating it from the high-energy regions (Figure 9E,F). This confirms that the ligand-bound complex converged to a well-defined, thermodynamically stable conformational state within 100 ns. Collectively, MD simulation analyses demonstrate that the complex of compound **1** and 23S rRNA is thermodynamically stable throughout the 100 ns simulation, with a stable binding region located between two highly flexible regions. Through hydrophobic stacking interactions, ligand binding induces a more compact conformation, which given the inherently high flexibility of 23S rRNA, conformationally disrupts its normal biological function and thereby exerts antibacterial activity.

### 2.16. Inhibitory Effect In Vitro Translation Analysis

This experiment aimed to verify whether compound **1** targets 23S rRNA to directly inhibit bacterial protein translation and modulate GTP hydrolysis. A commercial *Escherichia coli* in vitro coupled transcription–translation system was employed, with compound **1** supplemented at concentrations of 1 ×, 1/2 ×, and 1/4 × MIC. In the control group without compound-**1** treatment, functional 23S rRNA mediated peptide synthesis, enabling successful translation of luciferase. At 1/4 × MIC and 1/2 × MIC, trace amounts of compound **1** targeted partial 23S rRNA and interfered with peptide elongation, thus suppressing protein translation. The inhibited fraction of 23S rRNA failed to maintain normal GTP hydrolysis activity, resulting in a significant, dose-dependent reduction in both luciferase activity and GTP hydrolysis levels. At 1 × MIC, compound **1** fully targeted 23S rRNA and completely blocked peptide synthesis. Accordingly, luciferase activity decreased to nearly zero (Figure 10A). The inactivated 23S rRNA could no longer activate GTPase, and GTP hydrolysis was restricted to a low basal spontaneous hydrolysis level (Figure 10B). The results indicate that compound **1** is capable of inhibiting bacterial protein translation.

## 3. Discussion

Vibriosis caused by *V. alginolyticus* severely damages mariculture and threatens public health, while extensive antibiotic abuse has led to widespread bacterial drug resistance, making it urgent to explore natural alternative bacteriostatic agents. Mangrove endophytic fungi are rich sources of bioactive metabolites yet lack systematic research on anti-vibrio molecular mechanisms. In this study, *P. janthinellum* D131 was isolated from medicinal mangrove Kandelia candel in Qinzhou Bay. Five secondary metabolites were purified through rice solid-state fermentation and chromatographic separation. Antibacterial evaluation verified that **1** exerted superior inhibitory activity against *V. alginolyticus*, with a lower MIC (0.78 μg/mL) than chloramphenicol (1.56 μg/mL).

Integrated phenotypic experiments, multi-omics analysis, molecular docking, molecular dynamics simulations, and in vitro translation assays jointly revealed the antibacterial target and action pathway of compound **1**. Molecular docking revealed that compound **1** binds to the Domain II region of 23S rRNA in the 50S subunit. MD simulations further demonstrated that this binding site is not only thermodynamically stable but also induces a more compact conformation of the complex. Given the inherent flexibility of 23S rRNA, this binding restricts its spatial conformational changes and disrupts its normal function, thereby causing abnormalities in the 50S ribosomal subunit and preventing successful protein translation. This disturbance impairs bacterial growth and cellular homeostasis, triggers extensive intracellular ROS accumulation, damages membrane integrity, and eventually causes bacterial death.

This study is the reporting the potent inhibitory activity of **1** against *Vibrio alginolyticus*. Integrative omics analysis, molecular biology, and computational biology techniques were applied to fully elucidate the overall mechanism responsible for the excellent antibacterial activity of this compound. The research findings provide a feasible strategy for the subsequent development of this compound as a novel antibacterial agent for aquaculture, and promote the development and application of lead compounds in this field.

## 4. Materials and Methods

### 4.1. General Experimental Procedures

Instruments: HR-ESI-MS spectra were acquired on a Bruker solanX 70FT-MS (Bruker Corporation, Billerica, MA, USA). Semi-preparative high-performance liquid chromatography (HPLC) was performed on a Quiksep LC-050D system (H&E Corporation Limited, Beijing, China) using an ODS column (250 × 10 mm, 5 um, YMC, Kyoto, Japan). The NMR spectral data were recorded on JNM-ECZ600R spectrometers (JEOL, Tokyo, Japan, 600 MHz for ^1^H NMR and 150 MHz for ^13^C NMR). ECD spectra were obtained on a circular dichromatic spectrometer (Biologic, Seyssinet-Pariset, France, MOS-500). Optical rotations were obtained on a modular circular polarimeter (Anton Paar, Graz, Austria, MCP 5100). Silica gel (Qing Dao Hai Yang Chemical Group Co., Qingdao, China; 200–300 mesh) and octadecylsilyl silica gel (YMC, Kyoto, Japan; 50 µm) were utilized for column chromatography (CC). Other equipment included a thermostatted shaker (Shanghai Zhichu Instrument Co., Ltd., Shanghai, China, ZQWY-200S), constant-temperature incubator (Shanghai Yuzhuo Instrument Co., Ltd., Shanghai, China, SPX-150BIII), Büchi B-100 HB rotary evaporator (Büchi Labortechnik GmbH, Switzerland), ZF-8 UV detector (Shanghai Jiapeng Technology Co., Ltd., Shanghai, China), analytical balance (Kunshan Youkeweite Electronic Technology Co., Ltd., Jiangsu, China, LQ-C2003), and 101A-0 blast drying oven (Changzhou Xunsheng Instrument Co., Ltd., Jiangsu, China). Materials: Used in the study were analytical methanol, petroleum ether, ethyl acetate; HPLC-grade methanol/acetonitrile (Xilong Chemical, Shantou, China); 200–300 mesh silica gel, GF-254 TLC plates (Qingdao Marine Chemical, Qingdao, China); YMC ODS-A C18 column (Kyoto, Japan), Merck reversed-phase silica gel (Merck KGaA, Germany); PDA/PDB media; and deuterated DMSO and MeOH (Sangon Biotech, Shanghai, China). Other assay reagents were also prepared.

### 4.2. Fungal Material and Cultivation

Endophytic fungi were isolated from the roots of fresh and healthy *Kandelia obovata* mangroves collected from Maowei Sea, Qinzhou, China. *P. janthinellum* was identified via ITS sequence analysis. It showed 99.16% maximum similarity 99%. Its sequence was submitted to NCBI GenBank (accession PV834242). The strain voucher (CCTCC NO: M 2025732) was deposited in CCTCC (China Center for Type Culture Collection). The fungus was cultured on PDA and stored at −20 °C. PDA recipe: 200 g potato, 15 g agar, 20 g glucose, 1 L distilled water, initial pH 7.0. To prepare seed culture, fungal plugs were cultured in liquid medium at 28 °C, 180 rpm for 4 days until peak mycelial biomass. The culture liquid served as seed for bioactivity screening and metabolite separation. Full sequences and NCBI BLAST results are shown in the Supplementary Materials [23].

### 4.3. Chemical Investigation

*P. janthinellum* D131 was cultured via rice solid-state fermentation. A total of 300 flasks (200 g rice and 20 mL distilled water per flask) were sterilized at 121 °C for 30 min, inoculated with fungal seed liquid, and incubated at 25 °C in darkness for 35 days. Fermented substrates were repeatedly extracted with ethyl acetate, and the merged extracts were vacuum-concentrated to obtain 56.3 g of crude extract. The crude extract was subjected to silica gel column chromatography with a petroleum ether–ethyl acetate gradient elution, yielding six fractions (Fr.A–Fr.F). Fr.B was separated by C18 reversed-phase chromatography to produce 16 subfractions Fr.B.1-Fr.B.16, and Fr.B.7 was purified by semi-preparative HPLC (*v*/*v*, CH_3_CN/H_2_O, 67:33) to obtain compound **1** (22.8 mg). Fr.C was separated by C18 reversed-phase chromatography to produce 18 subfractions Fr.C.1-Fr.C.18. Subfraction Fr.C.4 was further purified using semi-preparative HPLC (*v*/*v*, CH_3_CN/H_2_O, 65:35) to obtain **2** (10.3 mg). Subfraction Fr.C.6 was further purified using semi-preparative HPLC (*v*/*v*, CH_3_CN/H_2_O, 68:32) to yield compound **3** (23.7 mg). Fr.D was separated by C18 reversed-phase chromatography to produce 23 subfractions Fr.D.1-Fr.D.23. Subfraction Fr.D.12 was further purified using semi-preparative HPLC (*v*/*v*, CH_3_CN/H_2_O, 70:30) to gain compound **4** (13.8 mg).

### 4.4. In Vitro Antibacterial Bioassay

Antimicrobial activity against *V. alginolyticus* was assessed using 96-well microtiter plates. Bacteria were cultured in TBS medium at 37 °C for 18 h with shaking, and then diluted to approximately 10^6^ CFU/mL. Compounds were dissolved in 1% DMSO. Each compound was tested in triplicate across a dilution series (100–0.0625 μg/mL). After inoculation, plates were incubated at 37 °C for 24 h, and optical density at 600 nm was measured in triplicate [24].

### 4.5. Growth Curves

To investigate the inhibitory effect of compound **1** on the proliferation of *V. alginolyticus*, mid-logarithmic phase bacterial suspension was diluted with fresh Tryptic Soy Broth supplemented with sodium chloride to a concentration of 10^6^ CFU/mL. Compound **1** was added to reach final concentrations of 1/8 × MIC, 1/4 × MIC, 1/2 × MIC, 1 × MIC, and 2 × MIC. Bacterial cultures without compound treatment were set as blank control groups [25]. All samples were incubated in a shaking incubator at 37 °C and 180 rpm. Samples were collected every 2 h to determine the optical density at 600 nm (OD_600_) and count viable colonies (CFU/mL). All OD_600_ and colony count data were normalized against the initial values at time zero.

### 4.6. Extracellular Protein and DNA Content Assays

*V. alginolyticus* suspensions (1 × 10^6^ CFU/mL) in PBS were treated with compound **1** at final concentrations of 2 ×, 4 ×, 8 ×, and 16 × MIC. After incubation at 37 °C for 4 h, the mixture was centrifuged (3500 rpm, 5 min, 4 °C) and the supernatant collected. DNA concentrations were then measured by microspectrophotometry, and protein levels were determined using a BCA assay kit (Labshark, Hunan Bkmam Holding Co., Ltd, Hunan, China) [26].

### 4.7. Detection of Reactive Oxygen Assays

Intracellular ROS levels were detected with Phygene ROS Assay Kit (PH0630-EO, Fuzhou Phygene Biotechnology Co., Ltd., Fujian, China). *V. alginolyticus* was cultured in TBS medium to OD_600_ = 0.1, pelleted and rinsed with pH 7.2 PBS. Cells were stained with 10 μM DCFDA for 1 h at 37 °C in darkness, washed, and resuspended in PBS. Samples were treated with compound **1** at 1 ×, 2 ×, and 4 × MIC (PBS as solvent control) and incubated another hour [27]. Fluorescence (Ex 485 nm/Em 535 nm) was measured on SpectraMax iD5 (Molecular Devices Shanghai Corporation, Shanghai, China). Data were normalized to viable cell counts as relative fluorescence units, with three biological replicates for each group.

### 4.8. Plasma Stability Analysis of Compound **1**

Fifty microliters of serially diluted compound-**1** solution was added into each well of a 96-well plate. 1% Triton X-100 served as the positive control and PBS buffer as the negative control. Subsequently, 150 μL red blood cell suspension was added to each well, followed by incubation at 37 °C for 1 h. After centrifugation, 100 μL supernatant from each well was transferred to a new 96-well plate, and the absorbance was measured at 540 nm using a microplate reader (Infinite M Nano, Tecan Group Ltd., Mannedorf, Switzerland) [28].

### 4.9. Biofilm Assays

Mid-log *V. alginolyticus* was diluted to 4 × 10^6^ CFU/mL in TSB and seeded into 96-well plates (200 μL/well). Compound **1** or 1% DMSO control was added, with PBS in edge wells to reduce evaporation. After static incubation at 37 °C for 24 h, unattached bacteria were washed off with PBS [29]. Dried biofilms were stained with 0.01% crystal violet, washed, dried, dissolved in 70% ethanol; absorbance at 595 nm was detected on a TECAN Infinite M Nano microplate reader.

### 4.10. DAPI and PI Fluorescence Staining

*V. alginolyticus* colonies were inoculated into TSB medium and incubated with shaking (180 rpm, 37 °C) for 6 h. Compound **1** was then added to a final concentration of 4 × MIC, followed by continued incubation under the same conditions for 4 h. Cells were harvested by centrifugation (4000 rpm, 5 min, 4 °C), washed twice with PBS, and resuspended in the same buffer. After dual staining with DAPI (10 μg/mL, 20 μL) and PI (20 μg/mL, 20 μL) in darkness for 30 min, samples were visualized and imaged via a laser scanning confocal microscope [30].

### 4.11. Scanning Electron Microscopy (SEM)

SEM was used to observe compound-**1**-triggered morphological changes in *V. alginolyticus* [31]. Bacteria cultured in TSB (37 °C, 200 rpm, 8 h, 1 × 10^8^ CFU/mL) were treated with compound **1** at 4 × MIC for another 4 h [31,32]. Cells were pelleted, PBS-washed, fixed overnight with 2.5% glutaraldehyde at 4 °C, ethanol-dehydrated, critical-point dried, and gold-sputtered. Cell morphology was imaged via Hitachi SEM, with untreated bacteria as negative control [33,34].

### 4.12. Transcriptome Analysis

Sterilized TBS medium was inoculated with *V. alginolyticus* and cultured at 37 °C, 180 rpm for 15 h to mid-log phase. Cultures were split into compound-treated (1 × MIC) and control groups, incubated for another 6 h, and then snap-frozen in liquid nitrogen [35]. Transcriptome sequencing was completed by Majorbio Bio-Pharm, Shanghai, China. Raw reads were quality-filtered and mapped to the reference genome via Hisat2 v2.0.5 for gene quantification. DEGs were screened and subjected to GO and KEGG functional enrichment analyses.

### 4.13. Quantitative Real-Time PCR (qRT-PCR) Analysis

qRT-PCR was performed to verify differentially expressed genes (DEGs) screened by transcriptome analysis. Total RNA was extracted from compound-treated (1 × MIC) and untreated *V. alginolyticus* using the TR205 RNA kit, and its concentration, purity, and integrity were determined via NanoDrop 2000 spectrophotometry and 1.5% agarose gel electrophoresis. First-strand cDNA was synthesized from 1 μg of total RNA using the FastKing cDNA Synthesis Kit with gDNA removal [36,37]. Seven DEG primers were designed via Oligo 7. qRT-PCR was conducted on a Bio-Rad CFX Connect System with SYBR Green qPCR premix in a 20 μL reaction system, containing 10 μL premix, 0.8 μL of each primer (10 μmol/L), 2 μL cDNA template, and 6.4 μL nuclease-free water [38,39].

### 4.14. Label-Free Quantitative Proteomics Analysis

Sterilized TBS medium was inoculated with *V. alginolyticus* and shaken at 37 °C, 180 rpm for 15 h to mid-log phase. Cultures were split equally into a compound-**1** treatment group (at a final concentration of 1 × MIC) and blank control group [40,41]. After 6 h incubation, cell pellets were snap-frozen in liquid nitrogen. Label-free quantitative proteomics was performed by Majorbio Bio-Pharm. Mass spectra were acquired in DDA mode, with MS/MS fragmentation of the top 10 precursor ions [42,43]. Raw data were processed using Progenesis 2.0, MASCOT 2.5, and SafeQuant for FDR correction and quantification, with three biological replicates.

### 4.15. Western Blot

Aseptically cultured mid-logarithmic *V. alginolyticus* (37 °C, 180 rpm, 15 h) was split into 10 mL subgroups: untreated control and treatments with compound **1** at 1/2 × MIC and 1/4 × MIC. After 6 h incubation, pellets were lysed via sonication (3 s pulse/4 s interval, 10 min on ice). Post-centrifugation supernatants were collected for Western blot. RplE primary antibody and goat anti-rabbit IgG secondary antibody were used. Bands were visualized with Biosharp ECL substrate; gray values were quantified by Image Lab 5.2.1 under auto-exposure Gray channel [44].

### 4.16. Docking

Molecular docking between compound **1** and 50S ribosomal (PDB: 6GC0) was first implemented in AutoDock 4.2.6. Protein–ligand interactions were visualized with UCSF Chimera and Discovery Studio [45]. The graphical user interface of AutoDock Tools (ADT) was utilized to prepare pdbqt files for the protein and ligand, and to construct the grid box. Grid maps were calculated via AutoGrid using a grid dimension of 60 × 60 × 60 grid points and a grid spacing of 0.375 Å. Both the protein and ligand were set as rigid structures during the docking process, and clustering analysis was performed on docking results with a positional root-mean-square deviation (RMSD) of 1.0 Å. The docking conformation with the lowest energy and optimal binding affinity was selected for alignment with the protein structure, followed by subsequent analysis using Biovia Discovery Studio 2025 visualization software. AutoDock Vina 1.2.7 was employed to further verify the predicted interactions between compound **1** and 50S ribosomal. Visualization of the results was conducted using PyMOL software (The PyMOL Graphics System, version 2.6.0; Schrödinger, LLC, New York, NY, USA) [46].

### 4.17. MD Simulations

Molecular dynamics simulations were performed using the GPU-accelerated GROMACS 2022.2 package. The 23S rRNA protein with PDB ID 6GC0 (the same as used in molecular docking) was employed as the receptor, and the ligand was the pdbqt file with the lowest free energy of −11.0 kcal/mol derived from docking. Ligand topology files were generated using the Sobtop 2026.1.16 and Multiwfn 2026.1.16 software packages [47]. Initial coordinates for MD simulations were taken from the protein–ligand complex structures obtained by molecular docking. Each complex was solvated in a cubic periodic box using the TIP3P water model. Sodium and chloride ions were added to neutralize the system charge and achieve a physiological ionic strength of 0.15 M. Energy minimization was carried out for 1000 steps using the conjugate gradient method to remove unfavorable atomic contacts [48]. The system was then sequentially equilibrated under NVT followed by NPT ensemble at 298.15 K and 1 bar. A 100 ns production MD simulation was performed on the equilibrated system. Trajectory analyses were conducted using built-in modules in GROMACS, and subsequent data processing and visualization were performed with Origin 2021 [49], PyMOL, and VMD software (Gromacs 2022). Free energy calculations and energy decomposition analyses were conducted using the gmx_MMPBSA program. Trajectory frames were extracted at 20 ps intervals starting from the system equilibration stage, with all other parameters kept at default settings.

### 4.18. In Vitro Transcription–Translation Assay

This experiment utilized the PURExpress^®^ In Vitro Protein Synthesis Kit from New England Biolabs (NEB). The total reaction volume was 25 μL, consisting of 5 μL S30 extract and 20 μL mixture. The mixture contained 0.1 mM amino acids, 10 μL S30 Premix [50], 1 μg luciferase plasmid pBESTluc, and serially diluted compound **1** or solvent control. The reaction mixture was incubated at 37 °C for 1 h, followed by incubation on ice for 5 min to terminate the reaction. Functional 23S rRNA facilitates polypeptide synthesis and continuously stimulates GTPase to hydrolyze GTP into GDP with the release of inorganic phosphate (Pi). The added dye forms a green complex with Pi [51], and the absorbance at A630 nm was measured via a microplate reader to characterize the GTP hydrolysis level under different treatments. The in vitro translation assay was repeated with the addition of luciferin substrate. Synthesized luciferase catalyzes reactions with luciferin [52], and chemiluminescence signals were detected using a microplate reader. The luminescence intensity reflects the overall transcription–translation activity.

### 4.19. ECD Computational Methods

The Spartan 14 program (Wavefunction Inc., Irvine, CA, USA) was used for calculating Merck molecular force field (MMFF). Density functional theory (DFT) and time-dependent density functional theory (TDDFT) calculations were performed using the Gaussian 16 program package. The conformational search was performed by an MMFF model, and then the conformers with lower relative energies (<10 kcal/mol) were subjected to geometry optimization with the DFT method at the B3LYP/6-31G* level. Vibrational frequency calculations were conducted at the same level to evaluate their relative thermal (Δ*E*) and free energies (ΔG) at 298.15 K. To obtain the energies of these low-energy conformers, the geometry-optimized conformers were further calculated at the B3LYP/6-311++G(d,p) level using the SMD [22,53]. The ^13^C NMR chemical shifts in **1A** and **1B** were calculated using the GIAO method at the MPW1PW91/6-311+G(d,p) level with the PCM. The shielding constants (σ) were converted into chemical shifts (δ) by referencing to TMS at 0 ppm (δcal = σTMS − σcal), where the σTMS was the shielding constant of TMS calculated at the same level. The calculated chemical shifts were statistically compared with the experimental chemical shifts using the DP4+ method. The DP4+ probability and linear correlation coefficients (*R*^2^) were adopted for evaluation of the results [22,53].

## Figures and Tables

**Figure 2:**
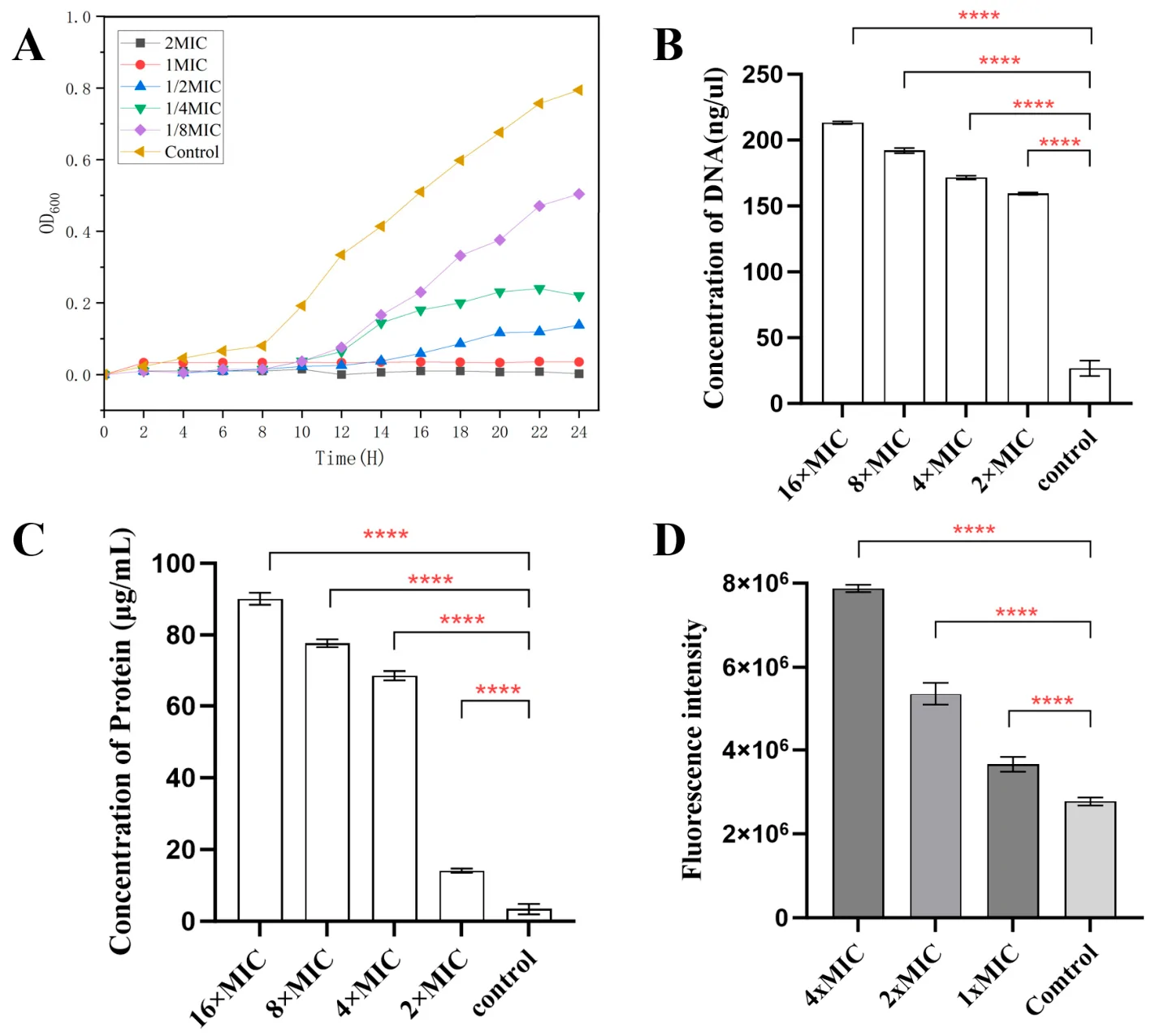
Effects of gradient concentrations of compound **1** on *V. alginolyticus*. (**A**) Growth curves of *V. alginolyticus* treated with serial doses of **1**. (**B**) Extracellular DNA content and (**C**) extracellular protein content of *V. alginolyticus* after 4 h treatment. (**D**) Intracellular ROS accumulation levels in *V. alginolyticus* following 4 h exposure to gradient concentrations of **1**. The data are plotted as mean SD of three replicates. ^****^p < 0.0001 (analyzed by an ordinary one-way ANOVA).

**Figure 3:**
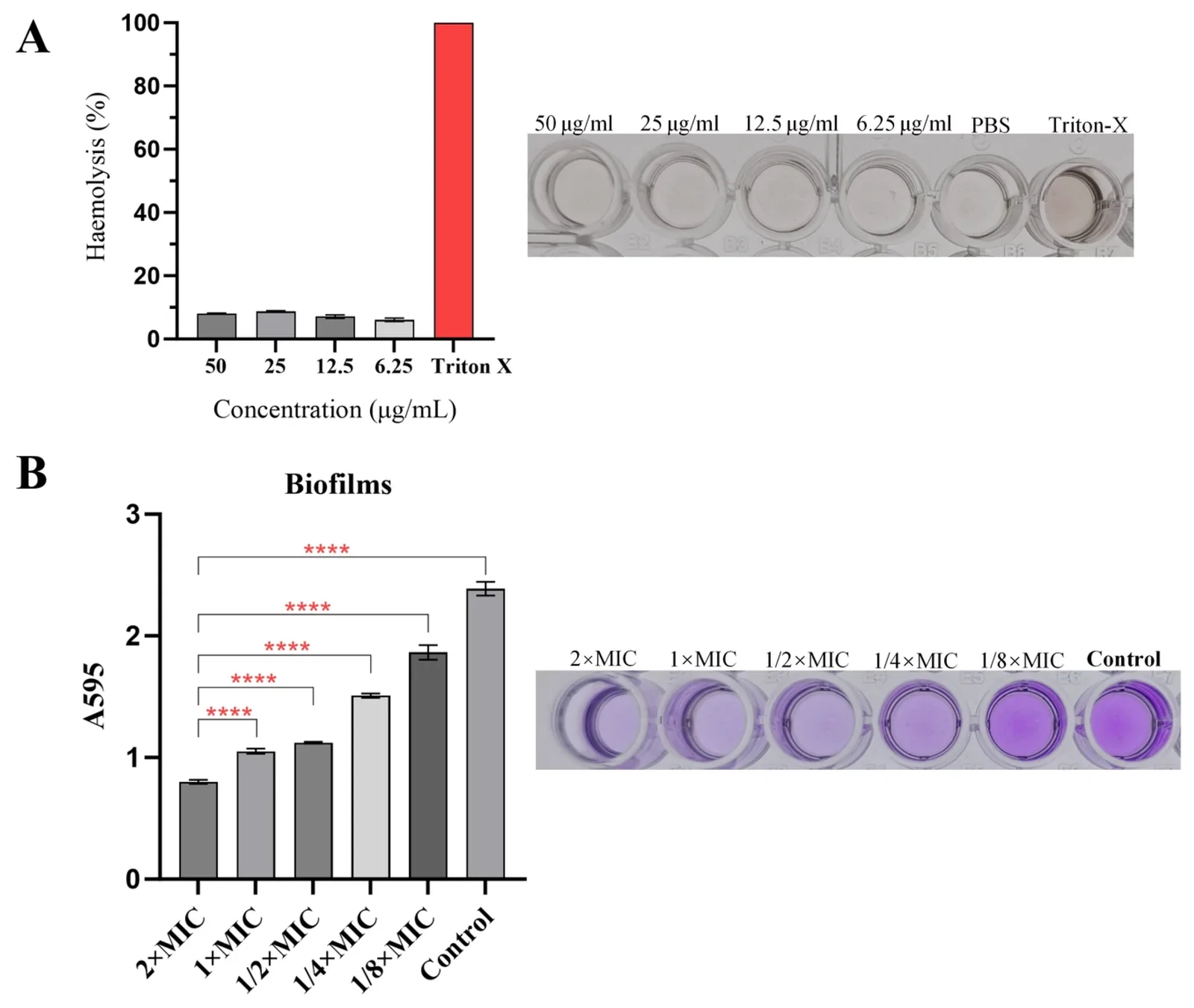
Anti-virulence and anti-biofilm activities of **1** against *V. alginolyticus*. (**A**) Hemolytic activity of *V. alginolyticus* treated with gradient concentrations of **1**. (**B**) Biofilm inhibition rate after 12 H co-incubation of *V. alginolyticus* with gradient concentrations of **1**. The data are plotted as mean SD of three replicates. ^****^p < 0.0001 (analyzed by an ordinary one-way ANOVA).

**Figure 4:**
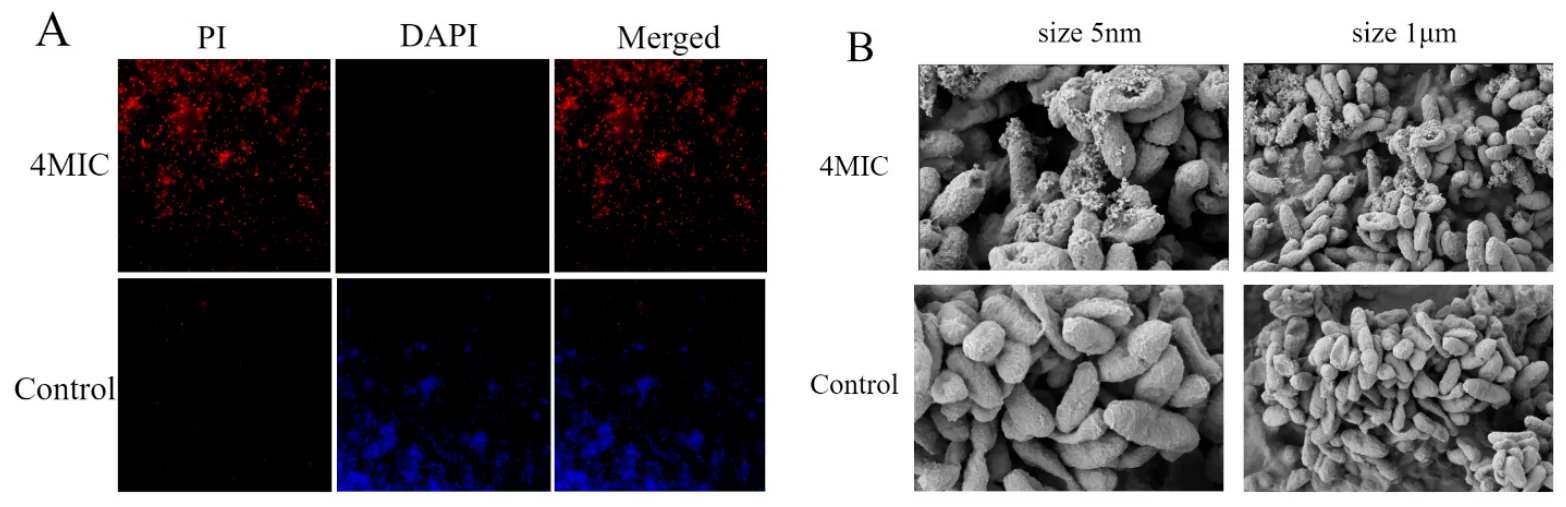
Cell membrane damage in *V. alginolyticus* induced by compound **1** at 4 × MIC. (**A**) Fluorescence staining images of *V. alginolyticus* cells co-stained with DAPI and PI. (**B**) Scanning electron microscopy (SEM) images showing morphological disruption of bacterial cells.

**Figure 5:**
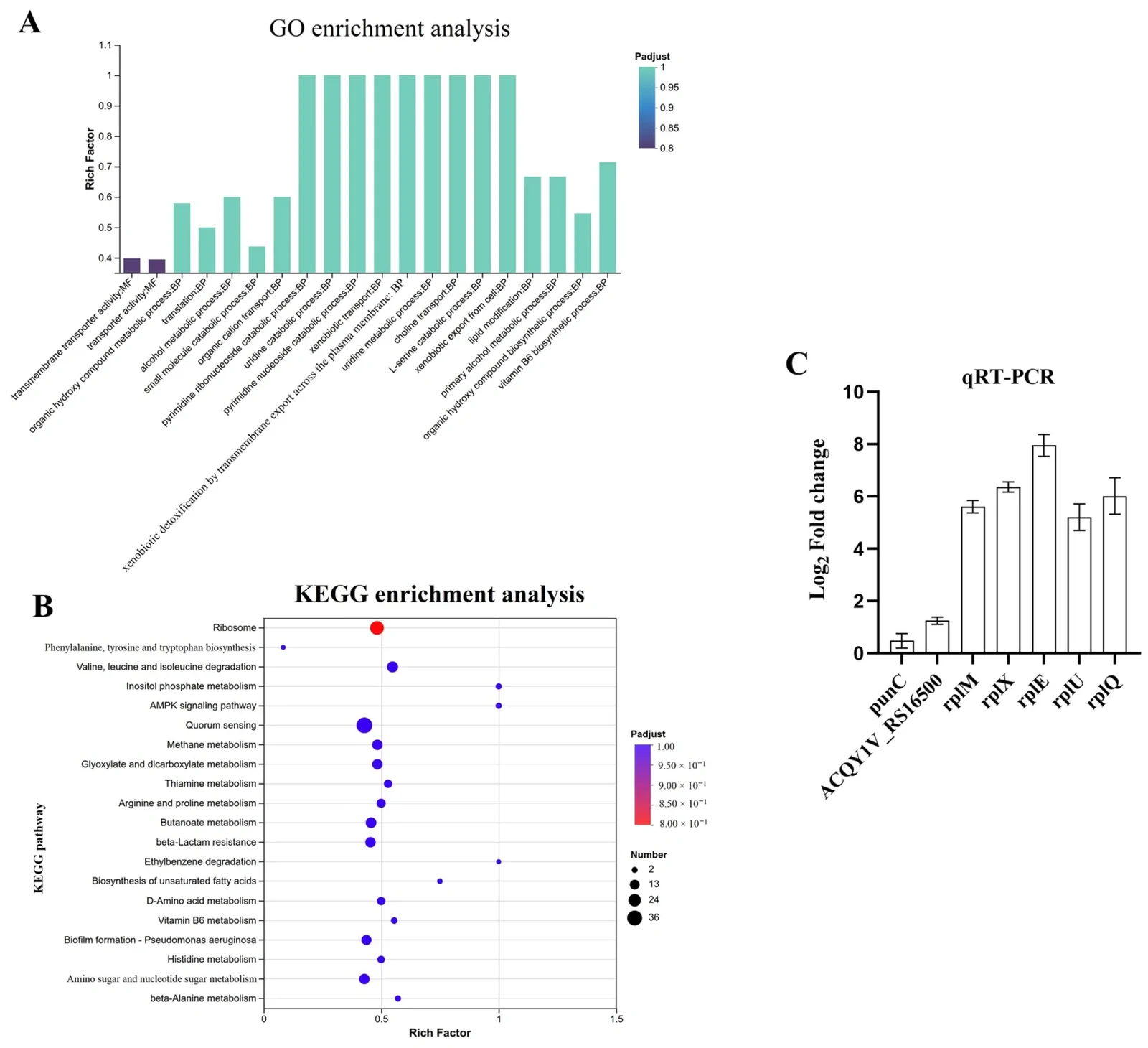
Transcriptomic profiling of *V. alginolyticus* treated with compound **1** at 1 × MIC. (**A**) GO enrichment analysis and (**B**) KEGG pathway enrichment analysis of differentially expressed genes. (**C**) qRT-PCR validation of seven representative differentially expressed genes under the same treatment condition.

**Figure 6:**
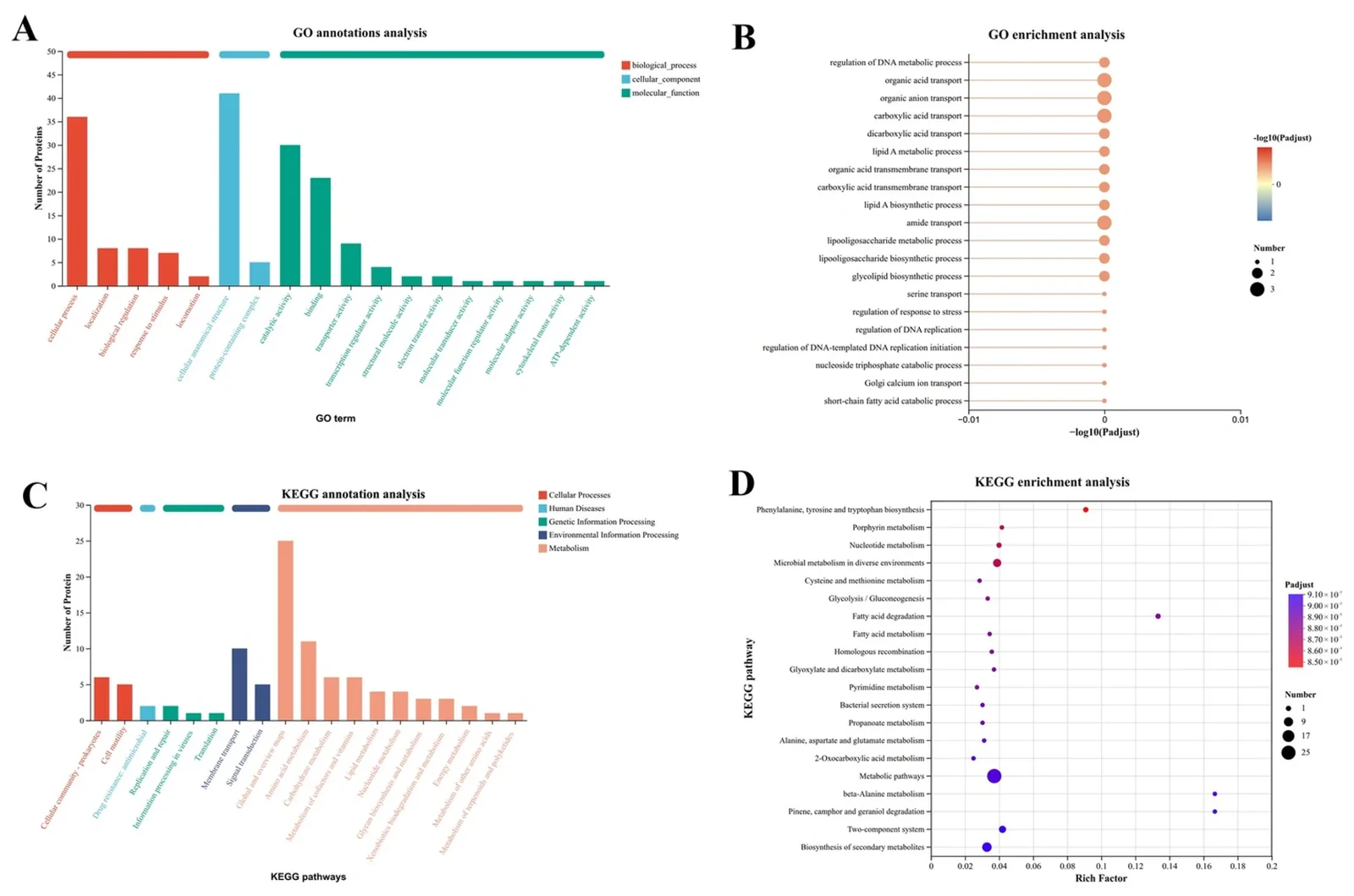
Label-free quantitative proteomic analysis of *V. alginolyticus* treated with compound **1** at 1 × MIC. (**A**) GO classification statistics and (**B**) GO enrichment analysis of differentially expressed proteins. (**C**) KEGG classification statistics and (**D**) KEGG pathway enrichment analysis of differentially expressed proteins.

**Figure 7:**
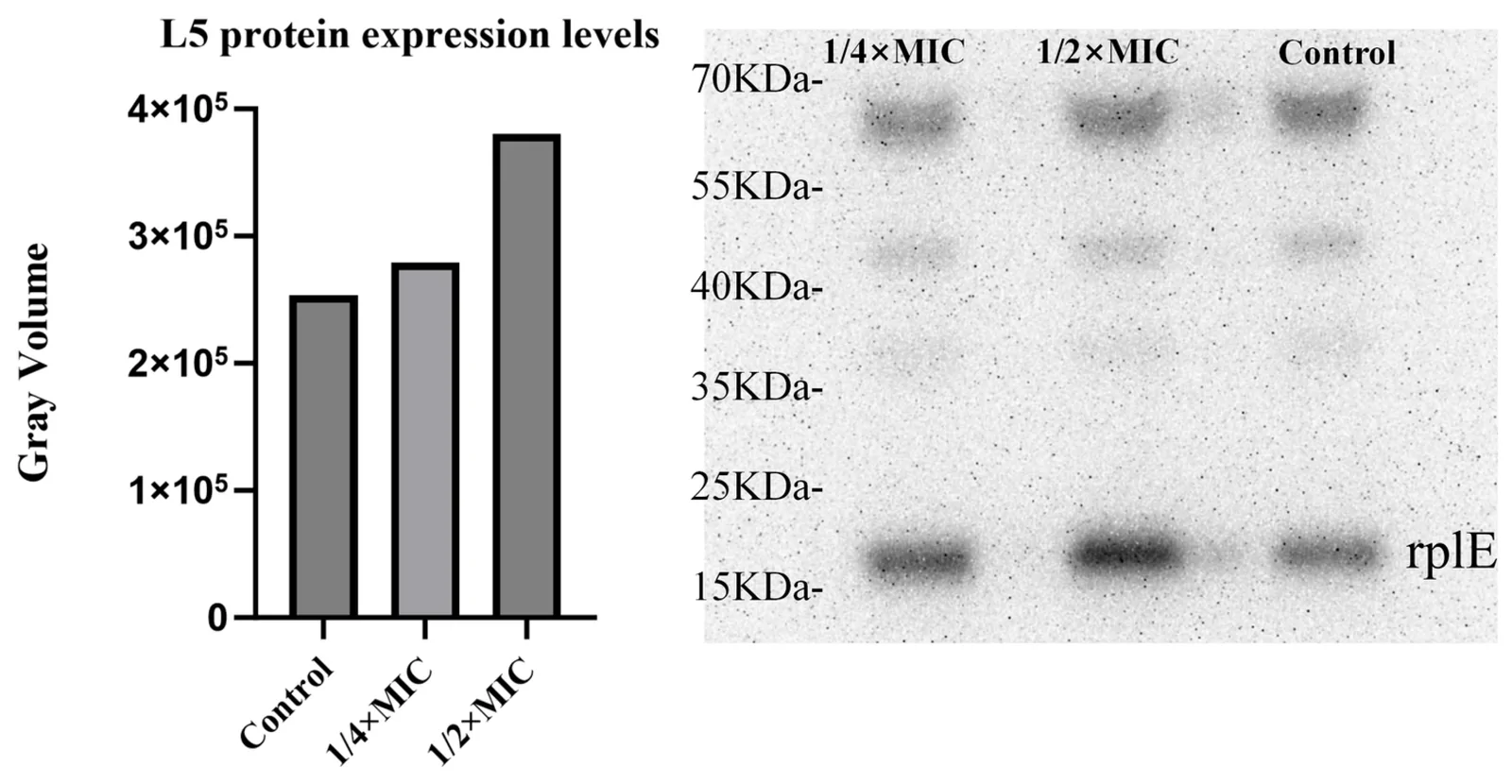
Verification of the ribosome-targeted inhibitory effect of **1**. Expression level of ribosomal protein L5 (*rplE*); (**left**): quantitative histogram, (**right**): Western blot band image.

**Figure 8:**
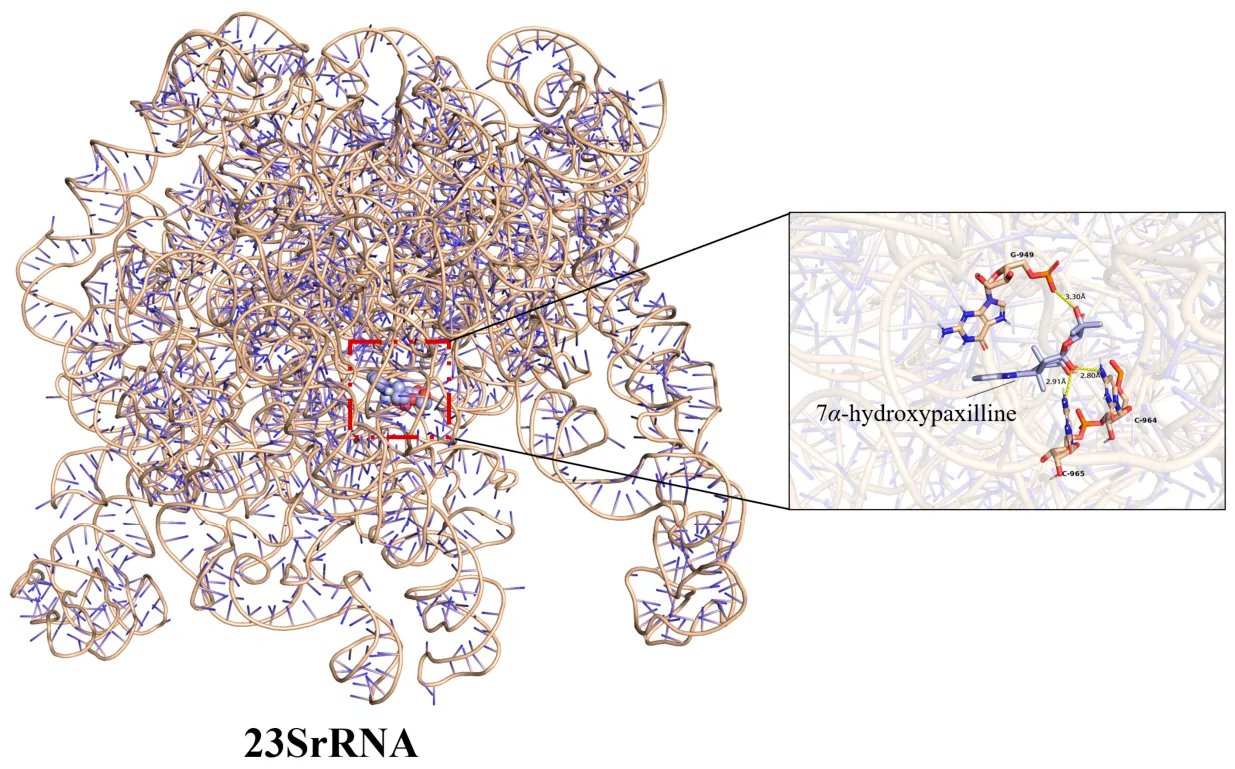
Molecular Docking of compound 1 with the 50S ribosomal subunit was performed to identify specific binding sites on 23S rRNA. PyMOL was used to visualize the molecular interactions. The inset shows the detailed binding mode of the small molecule. 7α-Hydroxypaxilline binds to residues G949 and C964 of 23S rRNA. Yellow dashed lines represent hydrogen bonds with distances of 3.30 Å, 2.91 Å, and 2.80 Å. Atoms are colored by element: nitrogen (N), blue; oxygen (O), red; phosphorus (P), orange.

**Figure 9:**
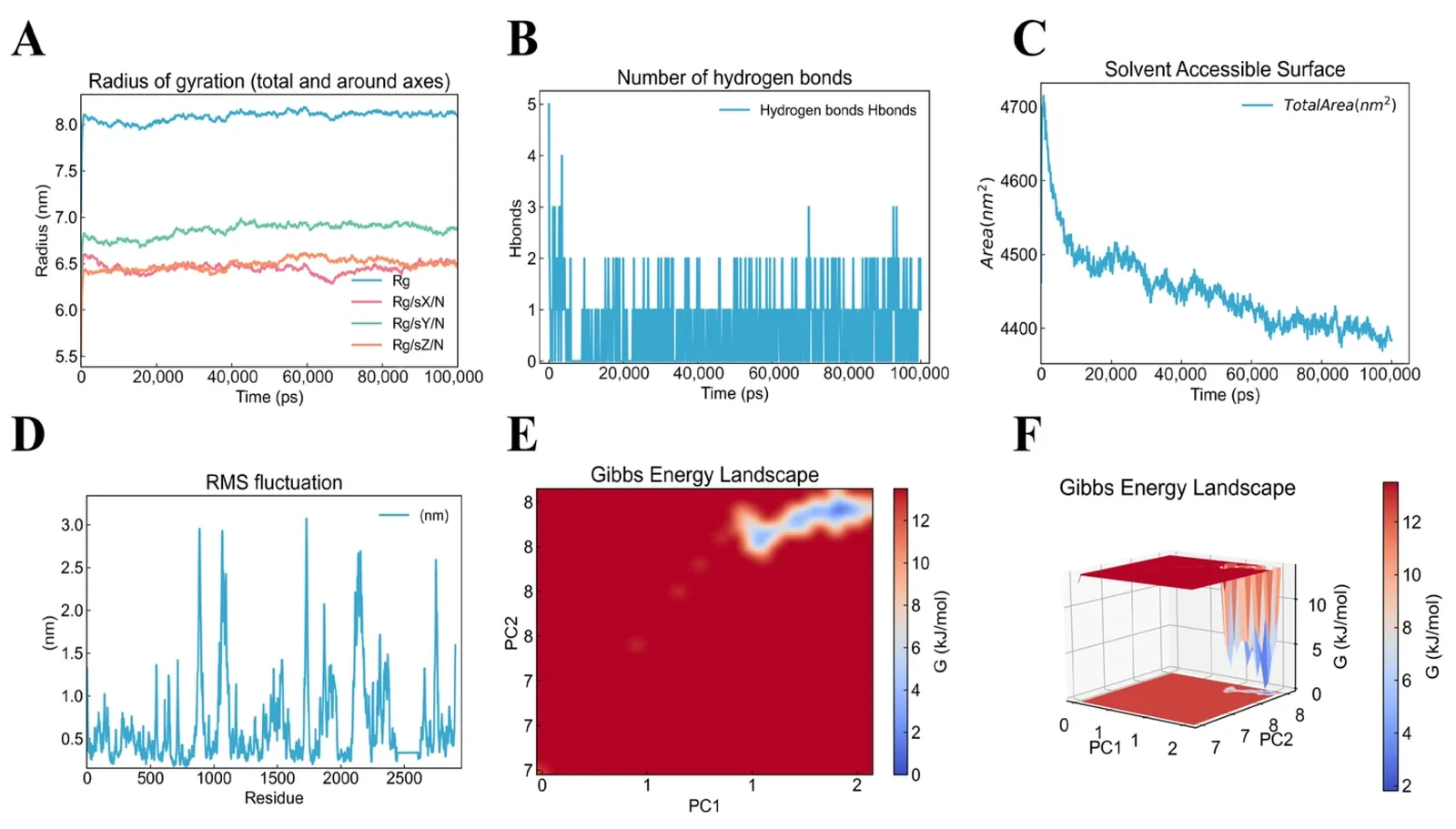
(**A**) Radius of gyration (Rg) profiles of the 23S rRNA (Rg_total, blue curve) and its principal axis components (RgX, red; RgY, green; RgZ, orange) over 100 ns MD simulations. (**B**) Hydrogen bond analysis: time evolution of hydrogen bonds between the ligand and 23S rRNA (blue curve), with the hydrogen bond criterion defined as a donor–acceptor (D–A) distance ≤ 0.35 nm and a D–H–A angle ≤ 30°. (**C**) Solvent-accessible surface area (SASA) of the 23S rRNA over the entire simulation period. (**D**) Root-mean-square fluctuation (RMSF) of each nucleotide in the 23S rRNA, indicating residue-level flexibility. (**E**) Two-dimensional Gibbs free energy landscape plotted against the first two principal components (PC1 and PC2). (**F**) Three-dimensional representation of the Gibbs free energy landscape, showing conformational free energy minima regions.

**Figure 10:**
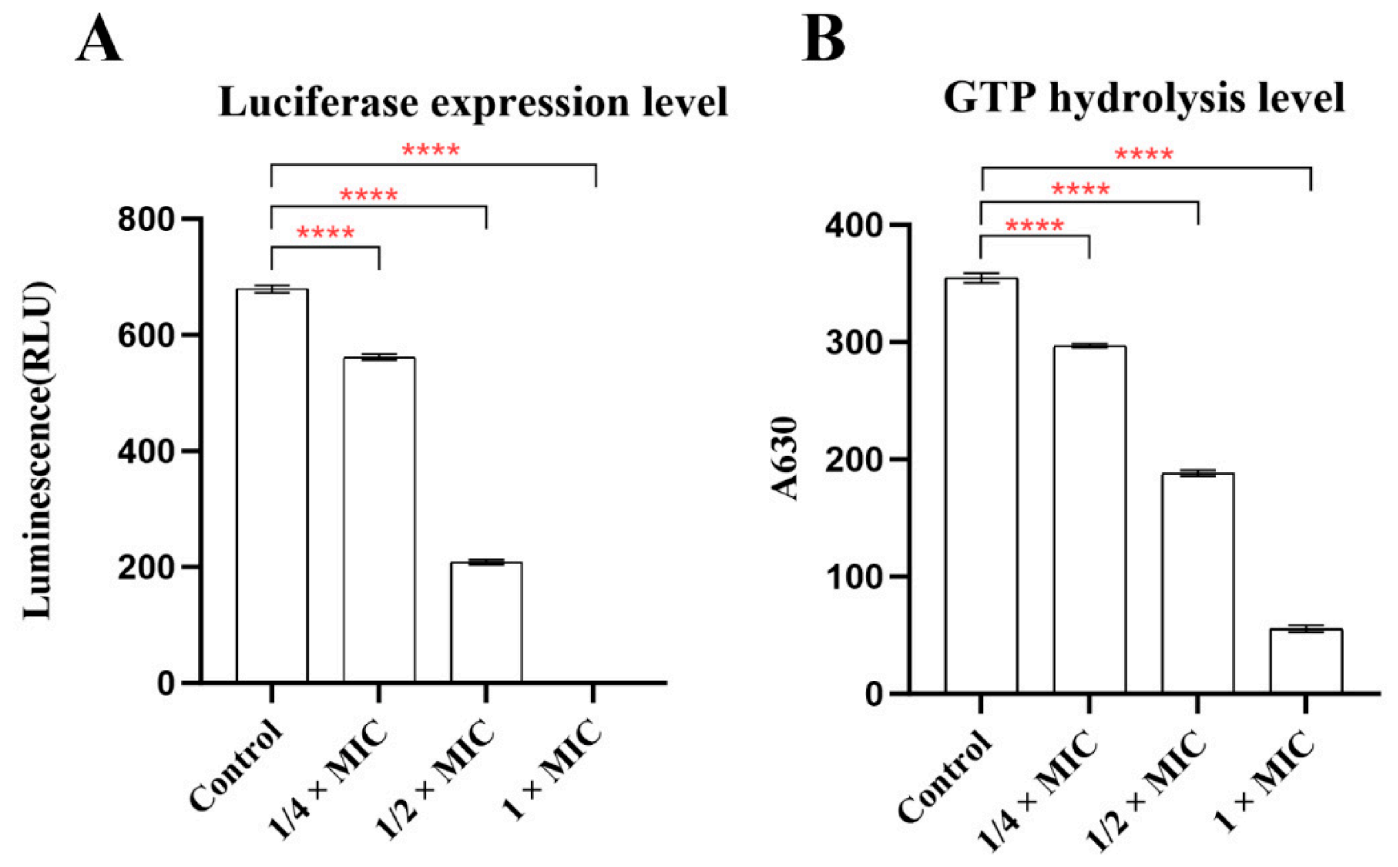
(**A**) Bar chart showing the chemiluminescence intensity of in vitro translation-inhibited luciferase activity. The luciferase translated in vitro was reacted with luciferin substrate, and the chemiluminescence intensity was measured. (**B**) Bar chart showing GTP hydrolysis levels. During translation, GTPase is activated to hydrolyze GTP into GDP and inorganic phosphate (Pi); a dye was subsequently added to react with Pi to form a green complex, and the absorbance at 630 nm (A_630_) was measured using a microplate reader. The data are plotted as mean SD of three replicates. ^****^p < 0.0001 (analyzed by an ordinary one-way ANOVA).

## Data Availability

The original contributions presented in this study are included in the article and Supplementary Materials. Further inquiries can be directed to the corresponding authors.

## Supplementary Materials

The following supporting information can be downloaded at: https://www.mdpi.com/article/10.3390/md24100334/s1, Table S1: The NMR data of **1** (DMSO-*d*_6_, *δ* in ppm, *J *in Hz) and 7*α*-hydroxypaxilline (CDCl_3_, *δ* in ppm, *J* in Hz); Table S2: The NMR data of **2-4**, paspaline, paspalinine and 4a-demethylpaspaline-4a-carboxylic acid (DMSO-*d*_6_, *δ* in ppm, *J *in Hz).Table S3: Energies of the dominative conformers of compound **1**. Figure S1: Key HMBC, ^1^H−^1^H COSY, NOESY correlations, and experimental and calculated ECD spectra of compound **1**. Figure S2: Regression analyses of experimental versus calculated ^13^C NMR chemical shifts of model compounds **1A** and**1B**. Figure S3: DP4+ analysis result table of model compounds **1A** and**1B** (experimental for**1**, isomer 1 for**1A**, isomer 2 for**1B**). Figure S4: ^1^H NMR spectrum of compound**1**in DMSO-*d*_6_. Figure S5: ^13^C NMR spectrum of compound **1** in DMSO-*d*_6_. Figure S6: DEPT135 NMR spectra of compound **1 **in DMSO-*d*_6_. Figure S7: HSQC spectra of compound **1 **in DMSO-*d*_6_. Figure S8: HMBC spectra of compound **1 **in DMSO-*d*_6_. Figure S9: ^1^H−^1^H COSY spectra of compound **1** in DMSO-*d*_6_. Figure S10: NOESY spectra of compound **1** in DMSO-*d*_6_. Figure S11: (+)-HRESIMS spectrum of compound **1**. Figure S12: ^1^H NMR spectrum of compound **2** in DMSO-*d*_6_. Figure S13: ^13^C NMR spectrum of compound **2**in DMSO-*d*_6_. Figure S14: (+)-HRESIMS spectrum of compound **2**. Figure S15: ^1^H NMR spectrum of compound **3** in DMSO-*d*_6_. Figure S16: ^13^C NMR spectrum of compound **3** in DMSO-*d*_6_. Figure S17: (+)-HRESIMS spectrum of compound **3**. Figure S18: ^1^H NMR spectrum of compound **4** in DMSO-*d*_6_. Figure S19: ^13^C NMR spectrum of compound**4** in DMSO-*d*_6_. Figure S20: (–)-HRESIMS spectrum of compound **4**.
