# Supplementary material for: 7α-Hydroxypaxilline, a Natural Product Isolated from Mangrove Endophytic Fungi, Inhibits Protein Translation in Vibrio alginolyticus
Source: Mar Drugs. 2026 Sep 25;24(10):334. doi: 10.3390/md24100334 (PMC13707130; doi:10.3390/md24100334)
Supplement: Supplementary file 1 [file marinedrugs-24-00334-s001.zip › marinedrugs-4522767-supplementary.pdf]

Supplementary Material of Compounds 1-4

Contents

Table S1. The NMR data of **1** (DMSO-d_6, δ in ppm, J in Hz) and 7α-hydroxypaxilline ($CDCl_3$, δ in ppm, J in Hz)..**2**

Table S2. The NMR data of **2-4**, paspaline, paspalinine and 4a-demethylpaspaline-4a-carboxylic acid (DMSO-d_6, δ in ppm, J in Hz)..................3

Table S3. Energies of the dominative conformers of compound **1**.............................4

Figure S1. Key HMBC, ^{1}H–^{1}H COSY, NOESY correlations, and experimental and calculated ECD spectra of compound **1**...4

Figure S2. ^{1}H NMR spectrum of compound **1** in DMSO-d_6. 5

Figure S3. ^{13}C NMR spectrum of compound **1** in DMSO-d_6. 5

Figure S4. DEPT135 NMR spectra of compound **1** in DMSO-d_6..............................6

Figure S5. HSQC spectra of compound **1** in DMSO-d_6. 6

Figure S6. HMBC spectra of compound **1** in DMSO-d_6. 7

Figure S7. ^{1}H–^{1}H COSY spectra of compound **1** in DMSO-d_6..................................7

Figure S8. NOESY spectra of compound **1** in DMSO-d_6..8

Figure S9. (+)-HRESIMS spectrum of compound **1**. 8

Figure S10. ^{1}H NMR spectrum of compound **2** in DMSO-d_6. 9

Figure S11. ^{13}C NMR spectrum of compound **2** in DMSO-d_6. 9

Figure S12. (+)-HRESIMS spectrum of compound **2**. 10

Figure S13. ^{1}H NMR spectrum of compound **3** in DMSO-d_6. 10

Figure S14. ^{13}C NMR spectrum of compound **3** in DMSO-d_6. 11

Figure S15. (+)-HRESIMS spectrum of compound **3.** 11

Figure S16. ^{1}H NMR spectrum of compound **4** in DMSO-d_6. 12

Figure S17. ^{13}C NMR spectrum of compound **4** in DMSO-d_6. 12

Figure S18. (–)-HRESIMS spectrum of compound **4**..13

Table S1. The NMR data of **1** (DMSO-d_6, δ in ppm, J in Hz) and 7α-hydroxypaxilline ($CDCl_3$, δ in ppm, J in Hz).

No.	Compound **1**		7α-hydroxypaxilline [1]
	δ_C	δ_H	δ_H
1-NH		10.76 (1H, s)	7.68 (1H, s)
2	152.7, C		
3	50.4, C		
4	42.6, C		
5	25.3, CH_2	2.51 (1H, m)<br>1.83 (1H, m)	2.68 (1H, m)<br>1.43 (1H, m)
6	35.1, CH_2	2.18 (1H, m)<br>2.13 (1H, m)	2.07 (1H, m)<br>2.25 (1H, m)
7	93.8, C		
9	78.1, CH	4.16 (1H, s)	4.30 (1H, s)
10	198.3, C		
11	120.5, CH	5.76 (1H, s)	5.82 (1H, s)
12	161.4, C		
13	76.1, C		
14	32.6, CH_2	1.85 (1H, m)<br>1.70 (1H, m)	1.98 (1H, m)<br>1.67 (1H, m)
15	21.0, CH_2	1.94 (1H, m)<br>1.64 (1H, m)	2.02 (1H, m)<br>1.98 (1H, m)
16	49.4, CH	2.77 (1H, m)	2.78 (1H, m)
17	26.9, CH_2	2.62 (1H, m)<br>2.33 (1H, m)	2.68 (1H, m)<br>2.39 (1H, m)
18	115.0, C		
19	124.5, C		
20	117.7, CH	7.27 (1H, t, 7.0)	7.38 (1H, m)
21	118.5, CH	6.90 (1H, t, 7.4)	7.01 (1H, m)
22	119.3, CH	6.94 (1H, t, 7.6)	7.01 (1H, m)
23	111.8, CH	7.27 (1H, t, 7.0)	7.20 (1H, m)
24	139.8, C		
25	16.2, CH_3	1.18 (3H, s)	1.10 (3H, s)
26	19.0, CH_3	0.93 (3H, s)	1.02 (3H, s)
27	71.1, C		
28	26.2, CH_3	1.19 (3H, s)	1.19 (3H, s)
29	26.1, CH_3	1.26 (3H, s)	1.32 (3H, s)
7-OH			3.62 (1H, s)
13-OH		5.03 (1H, s)	3.92 (1H, s)
27-OH		4.32 (1H, s)	3.80 (1H, s)

Table S2. The NMR data of **2-4**, paspaline, paspalinine and 4a-demethylpaspaline-4a-carboxylic acid (DMSO-d_6, δ in ppm, J in Hz).

No.	**2**	**Paspaline** [2,3]	**3**	**Paspalinine** [4]	**4**	**4a-Demethylpaspaline-4a-carboxylic acid** [5]
	δ_C	δ_C	δ_C	δ_C	δ_C	δ_C
2	151.2, C	151.1, C	152.8, C	152.7, C	151.0, C	151.0, C
3	52.5, C	52.4, C	51.0, C	51.0, C	51.7, C	51.7, C
4	40.1, C	39.4, C	39.2, C	39.2, C	40.1, C	39.9, C
5	32.5, CH_2	32.4, CH_2	25.9, CH_2	26.1, CH_2	32.4, CH_2	32.4, CH_2
6	25.1, CH_2	25.0, CH_2	28.0, CH_2	28.0, CH_2	25.2, CH_2	25.2, CH_2
7	85.2, C	85.2, C	104.2, C	104.3, C	83.6, CH	83.7, CH
9	84.6, CH	84.5, CH	86.9, CH	87.1, CH	84.2, CH	84.3, CH
10	21.1, CH_2	21.0, CH_2	197.0, C	196.6, C	22.8, CH_2	22.8, CH_2
11	37.2, CH_2	37.2, CH_2	116.8, CH	116.8, CH	35.3, CH_2	35.3, CH_2
12	36.1, C	36.0, C	169.7, C	169.5, C	47.3, C	47.3, C
13	45.9, CH	45.9, CH	76.0, C	76.0, C	46.0, CH	46.1, CH
14	21.5, CH_2	21.5, CH_2	31.8, CH_2	32.0, CH_2	24.4, CH_2	21.6, CH_2
15	24.5, CH_2	24.5, CH_2	21.0, CH_2	21.0, CH_2	24.8, CH_2	24.8, CH_2
16	48.6, CH	48.5, CH	48.5, CH	48.5, CH	48.6, CH	48.7, CH
17	27.1, CH_2	27.0, CH_2	27.2, CH_2	27.3, CH_2	27.0, CH_2	27.0, CH_2
18	115.8, C	115.8, C	114.9, C	114.9, C	115.9, C	115.9, C
19	124.4, C	124.4, C	124.6, C	124.7, C	124.4, C	124.4, C
20	117.6, CH	117.5, CH	119.3, CH	119.1, CH	117.6, CH	117.7, CH
21	118.4, CH	118.3, CH	118.5, CH	118.4, CH	118.4, CH	118.5, CH
22	119.3, CH	119.3, CH	117.7, CH	117.6, CH	119.4, CH	119.4, CH
23	111.9, CH	111.8, CH	111.9, CH	111.8, CH	111.9, CH	111.9, CH
24	140.3, C	140.2, C	140.1, C	140.1, C	140.2, C	140.3, C
25	14.6, CH_3	14.5, CH_3	16.4, CH_3	16.3, CH_3	14.5, CH_3	14.5, CH_3
26	19.4, CH_3	19.4, CH_3	22.5, CH_3	22.5, CH_3	16.2, CH_3	16.2, CH_3
27	12.6, CH_3	12.6, CH_3	77.9, C	77.8, C	175.3, C	175.4, C
28	70.4, C	70.4, C	22.9, CH_3	22.8, CH_3	70.4, C	70.4, C
29	24.6, CH_3	24.6, CH_3	28.5, CH_3	28.6, CH_3	23.4, CH_3	24.5, CH_3
30	27.0, CH_3	26.8, CH_3			27.0, CH_3	27.0, CH_3

Table S3. Energies of the dominative conformers of compound **1**.

Compounds	No.	Structure	E (Hartree)	Population (%)
**Compound 1**	1	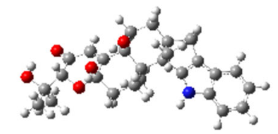	-1479.933308<br><br>12	86.17
	2	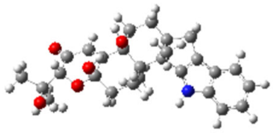	-1479.929718<br><br>53	0.57
	3	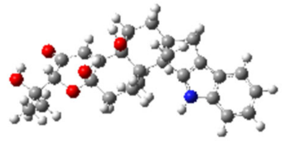	-1479.932361<br><br>16	13.26

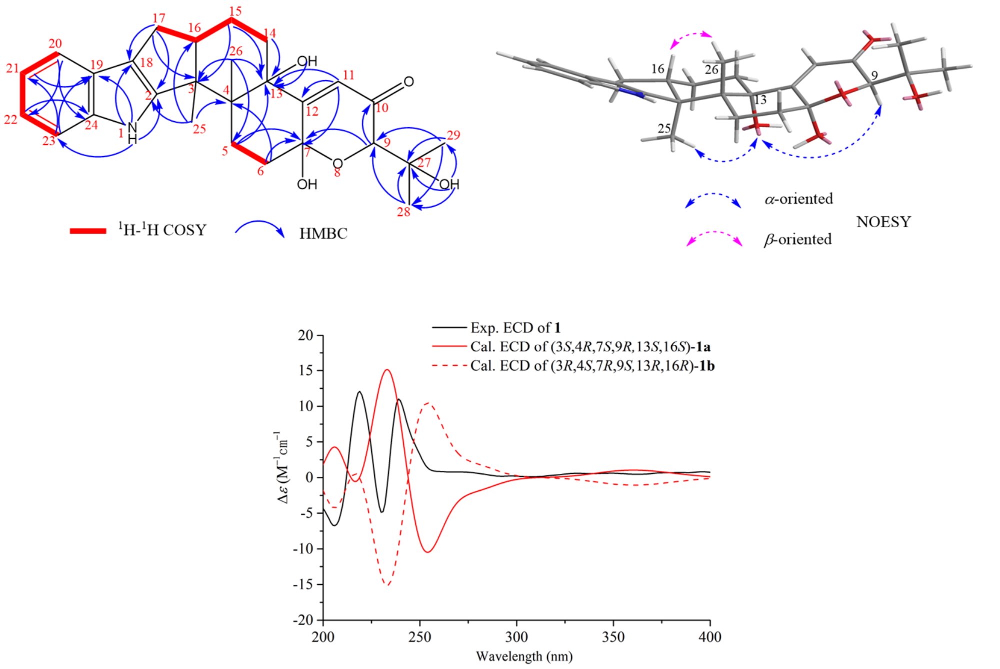

Figure S1. Key HMBC, ^{1}H−^{1}H COSY, NOESY correlations, and experimental and calculated ECD spectra of compound **1**.

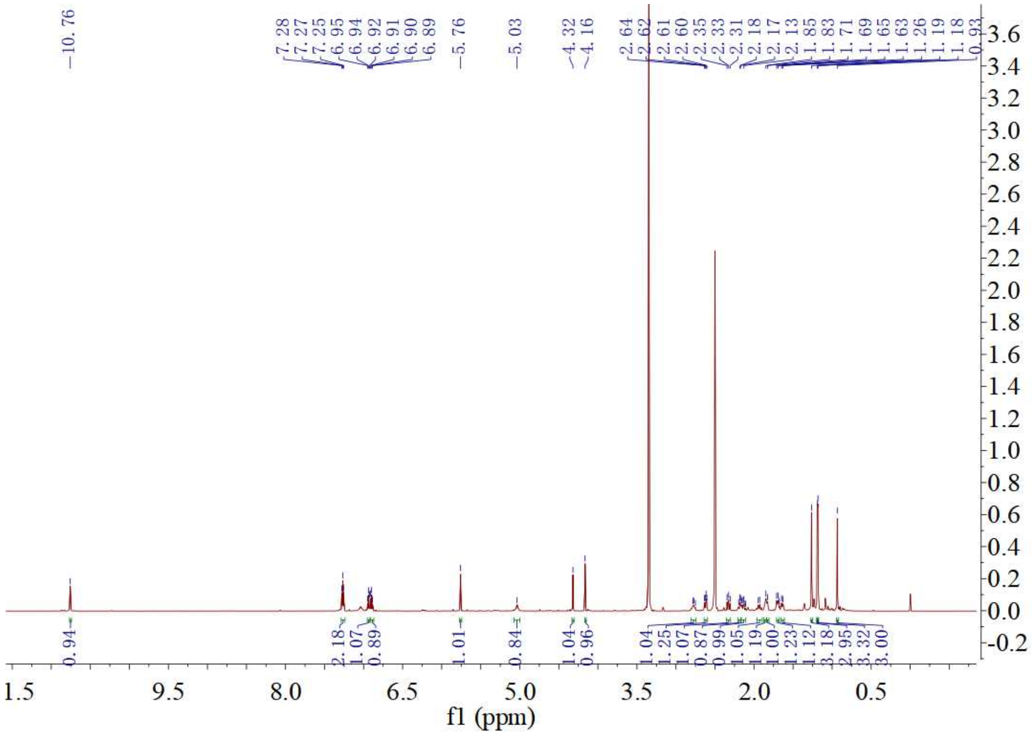

Figure S2. ^{1}H NMR spectrum of compound **1** in DMSO-d_6.

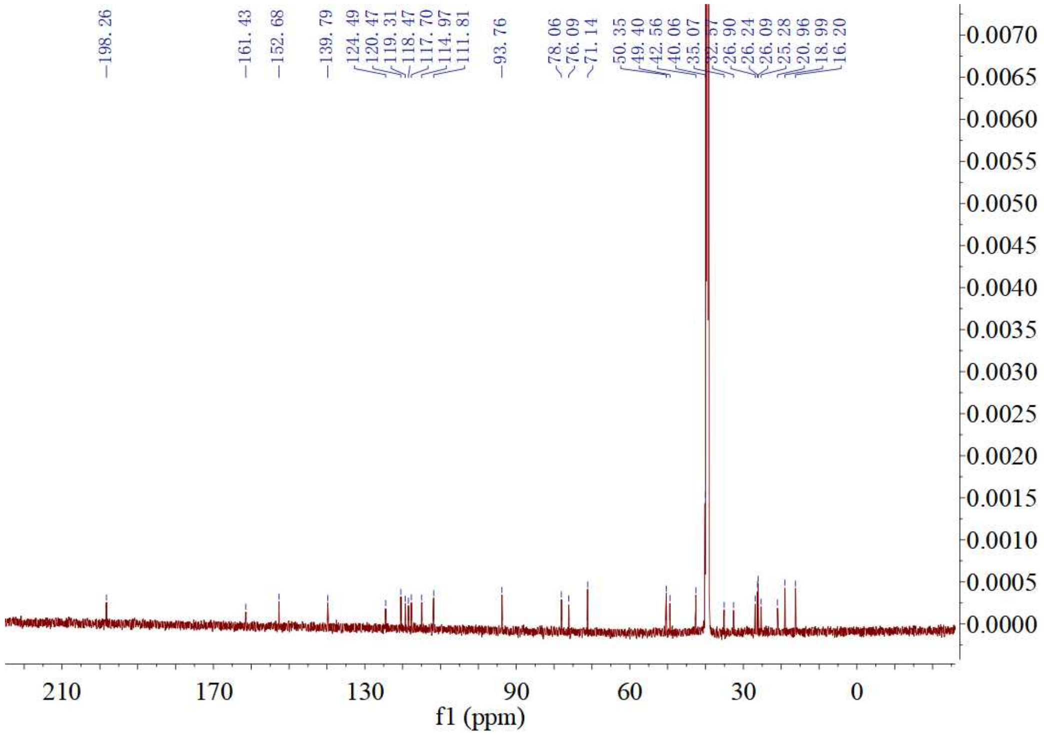

Figure S3. ^{13}C NMR spectrum of compound **1** in DMSO-d_6.

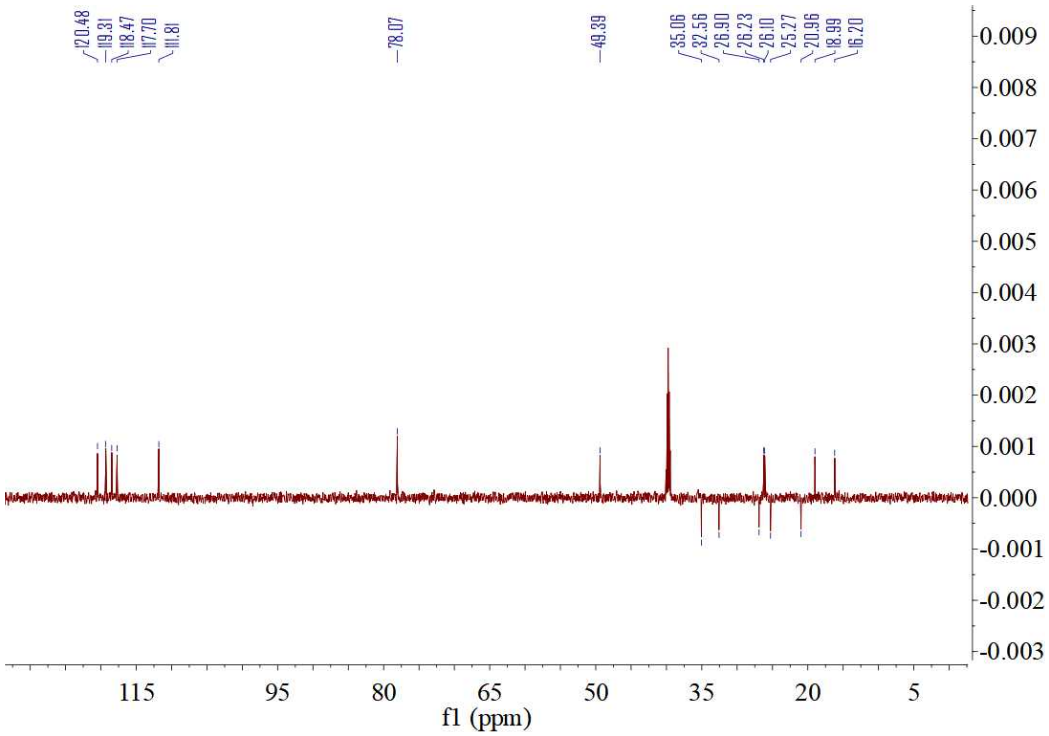

Figure S4. DEPT135 NMR spectra of compound **1** in DMSO-d_6.

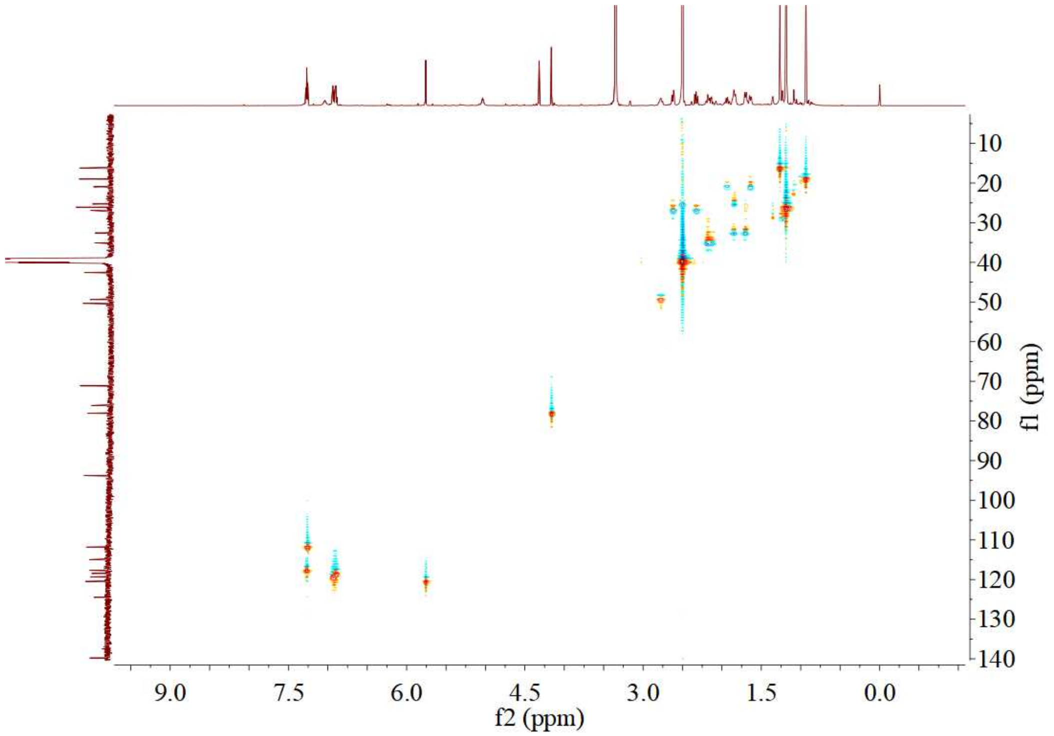

Figure S5. HSQC spectra of compound **1** in DMSO-d_6.

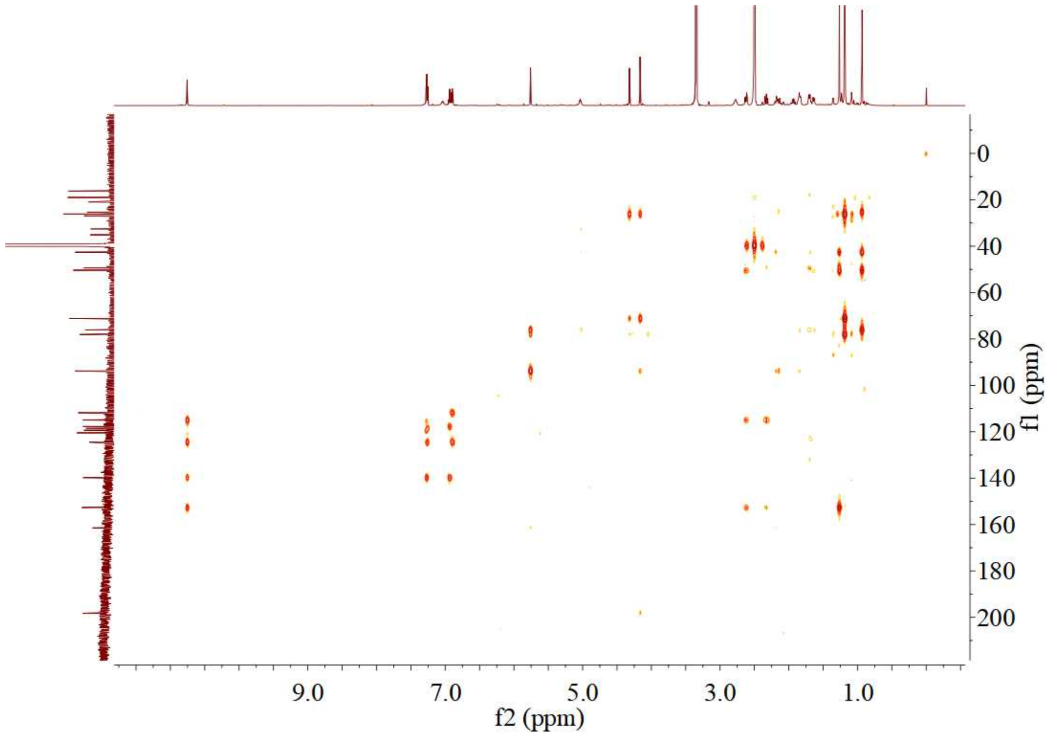

Figure S6. HMBC spectra of compound **1** in DMSO-d_6.

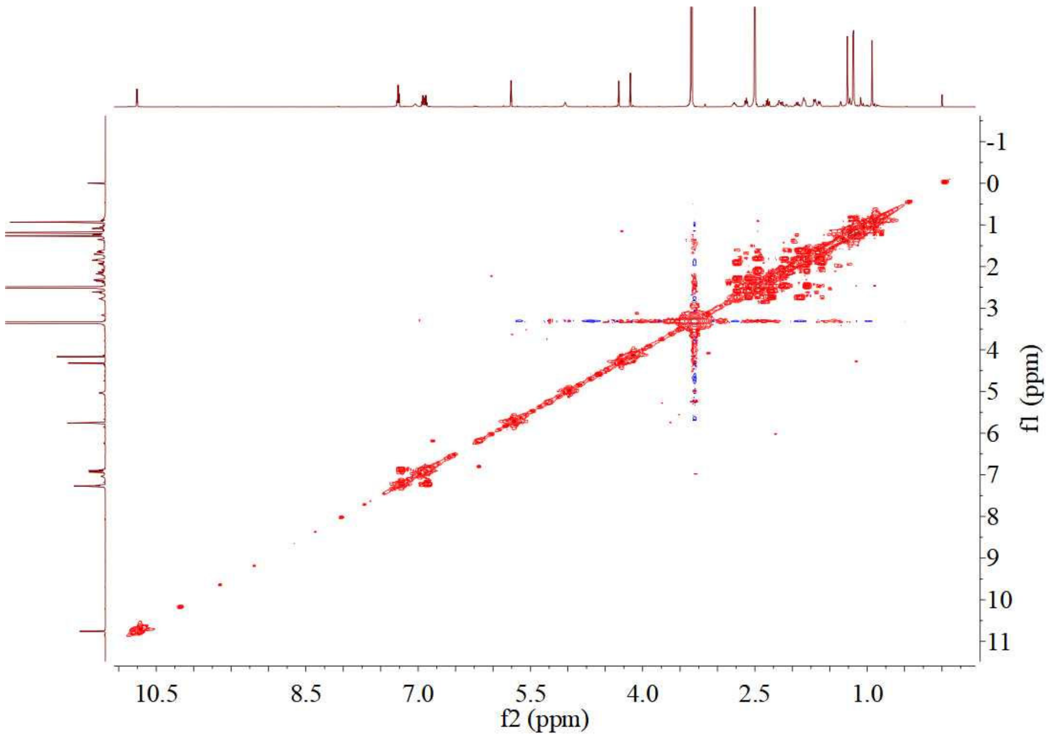

Figure S7. ^{1}H–^{1}H COSY spectra of compound **1** in DMSO-d_6.

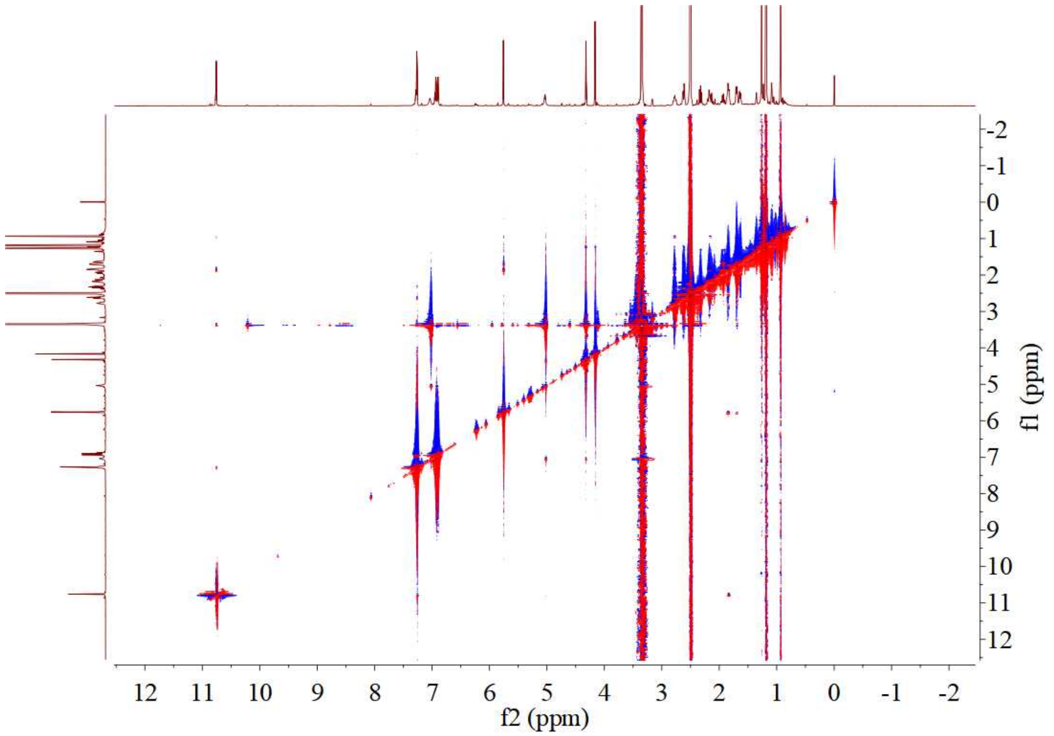

Figure S8. NOESY spectra of compound **1** in DMSO-d_6.

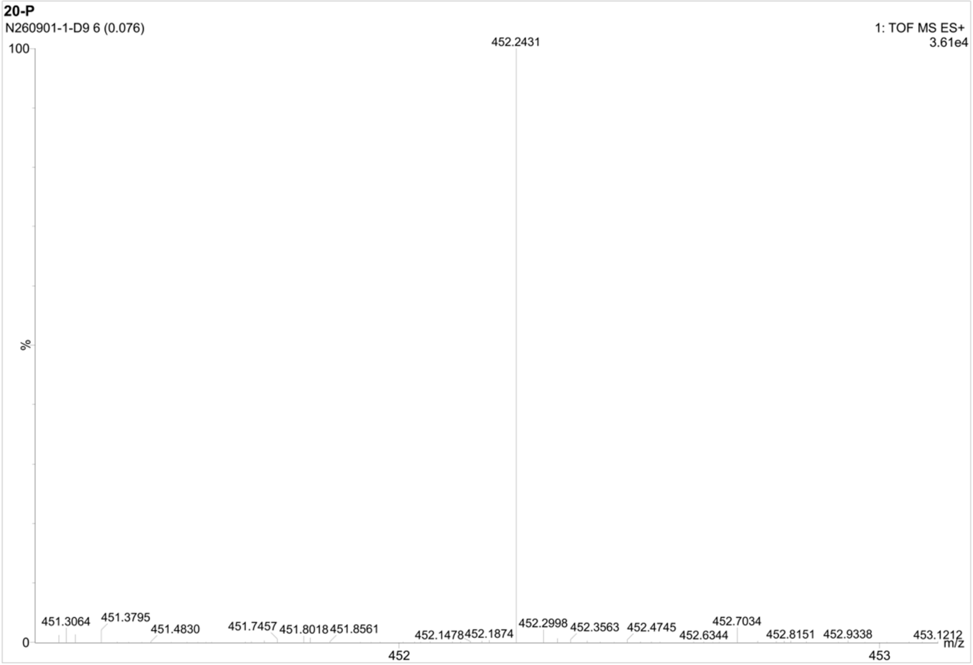

Figure S9. (+)-HRESIMS spectrum of compound **1.**

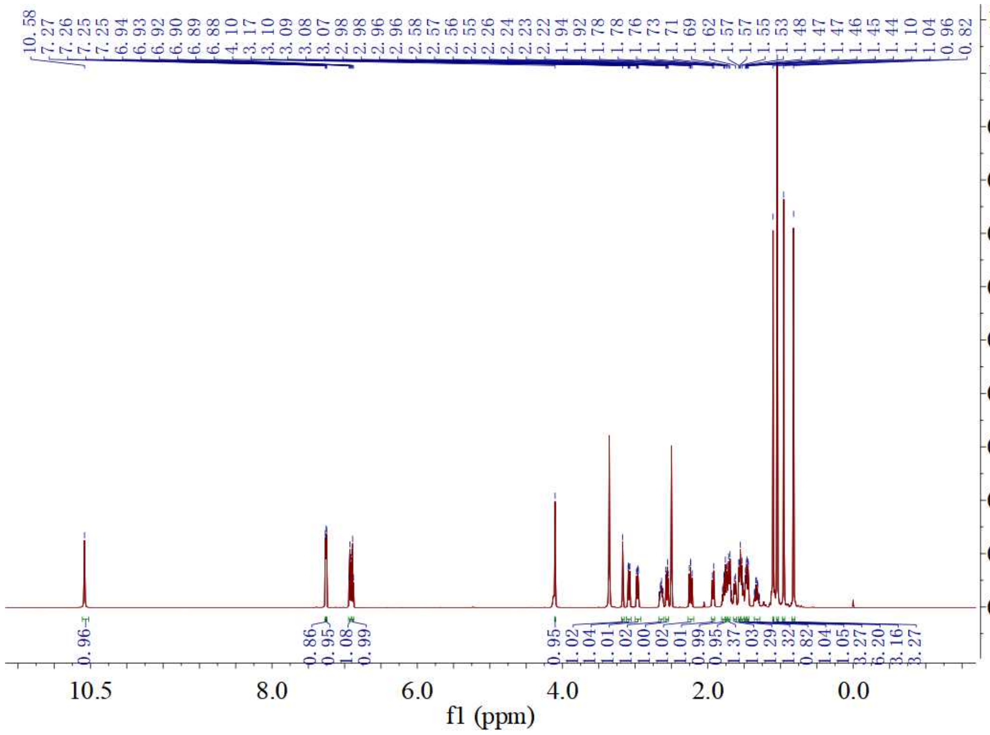

Figure S10. ^{1}H NMR spectrum of compound **2** in DMSO-d_6.

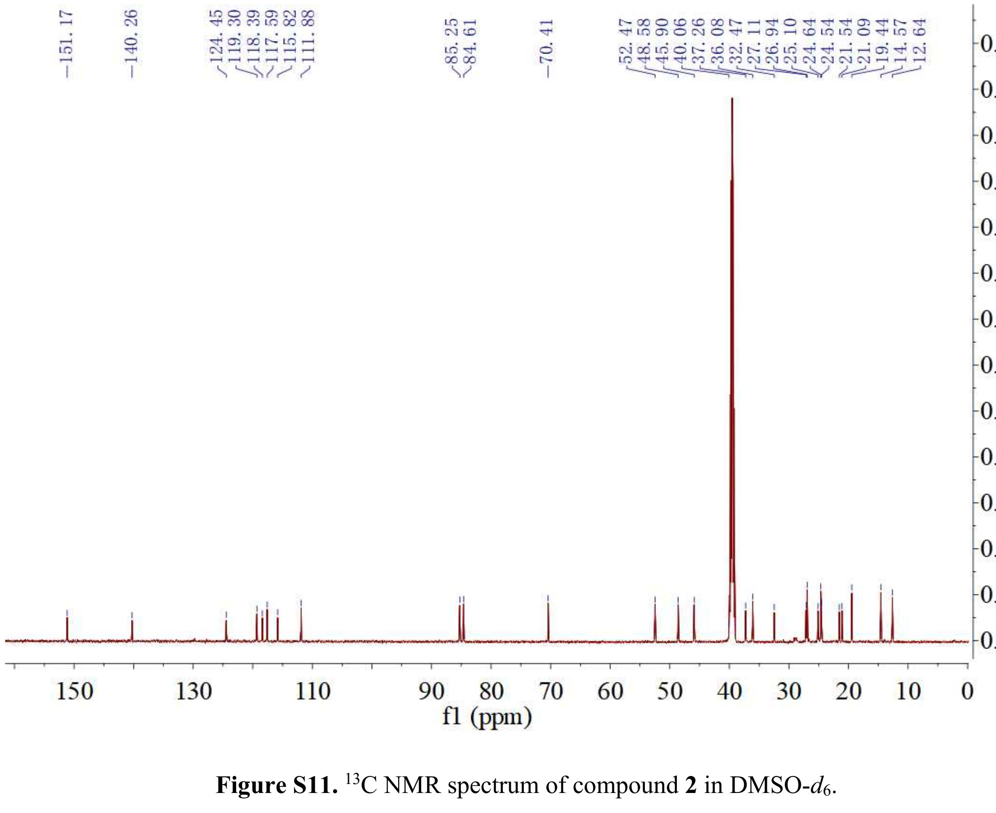

Figure S11. ^{13}C NMR spectrum of compound **2** in DMSO-d_6.

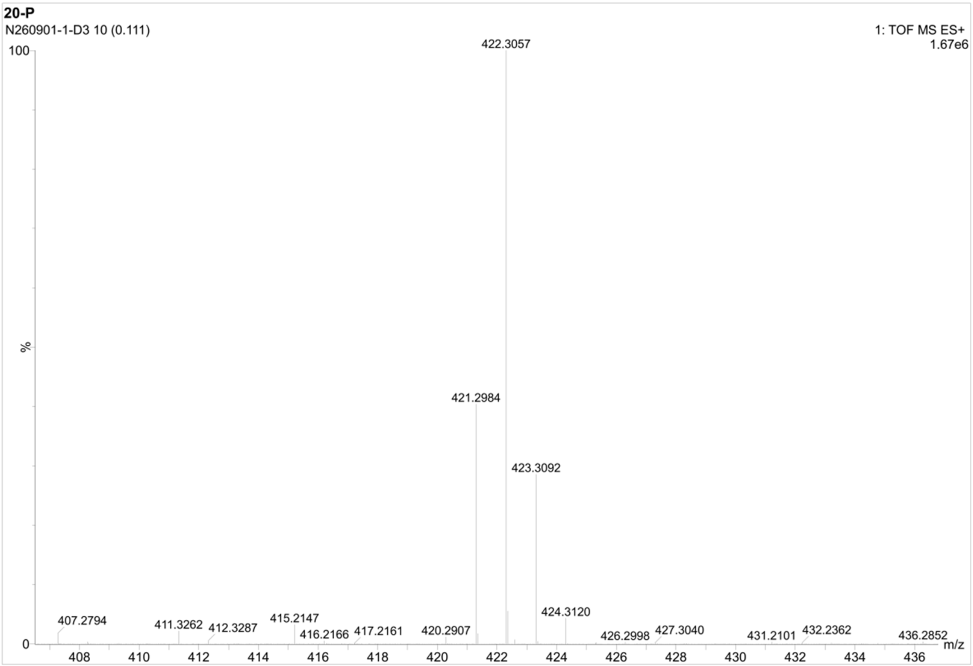

Figure S12. (+)-HRESIMS spectrum of compound **2.**

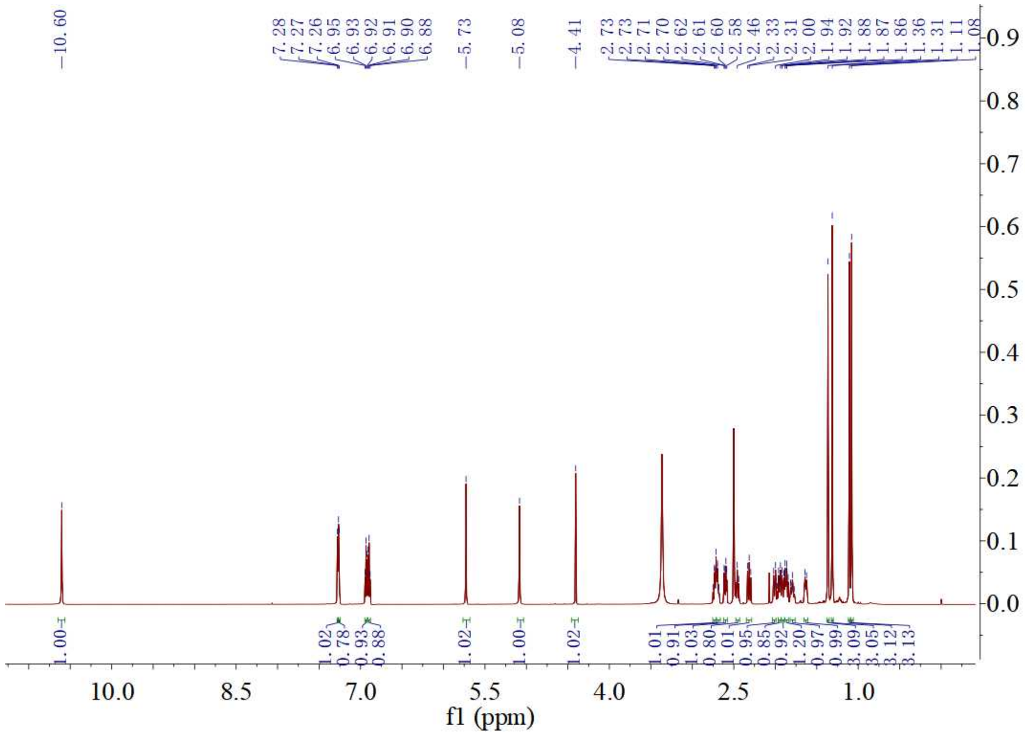

Figure S13. ^{1}H NMR spectrum of compound **3** in DMSO-d_6.

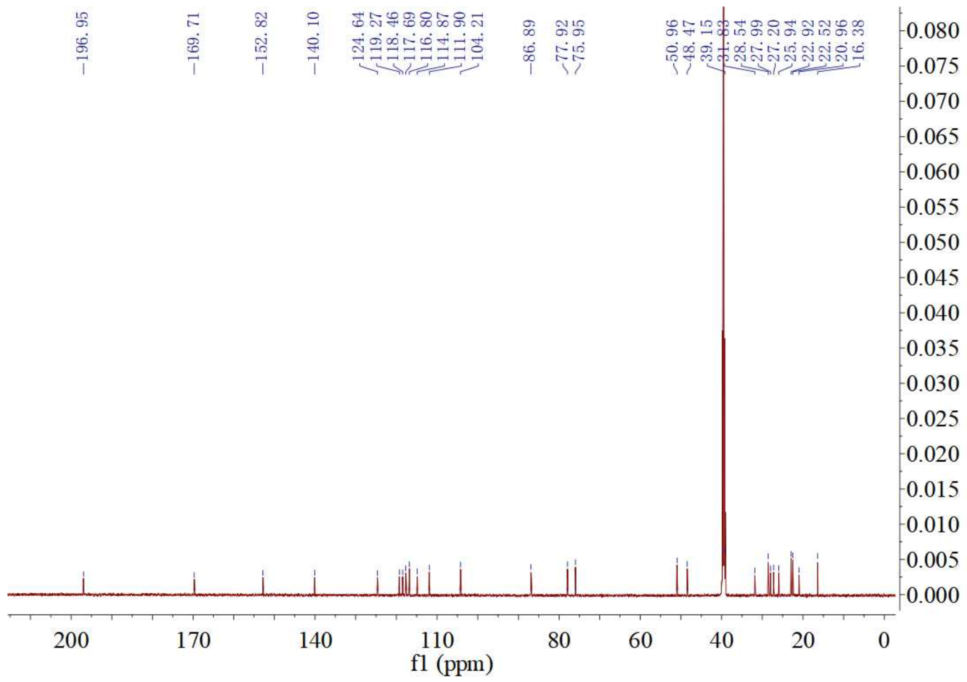

Figure S14. ^{13}C NMR spectrum of compound **3** in DMSO-d_6.

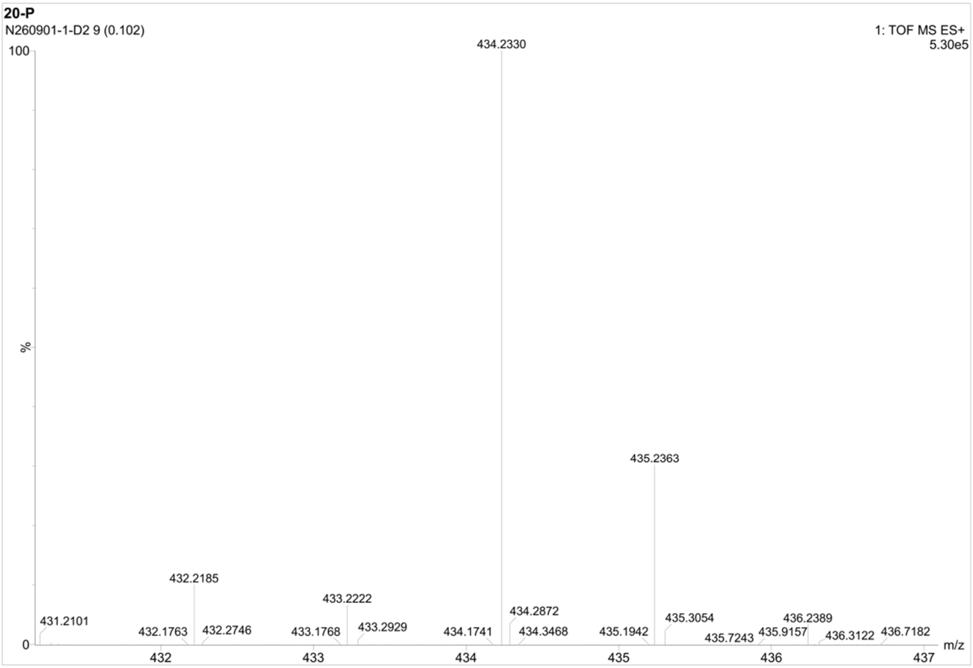

Figure S15. (+)-HRESIMS spectrum of compound **3.**

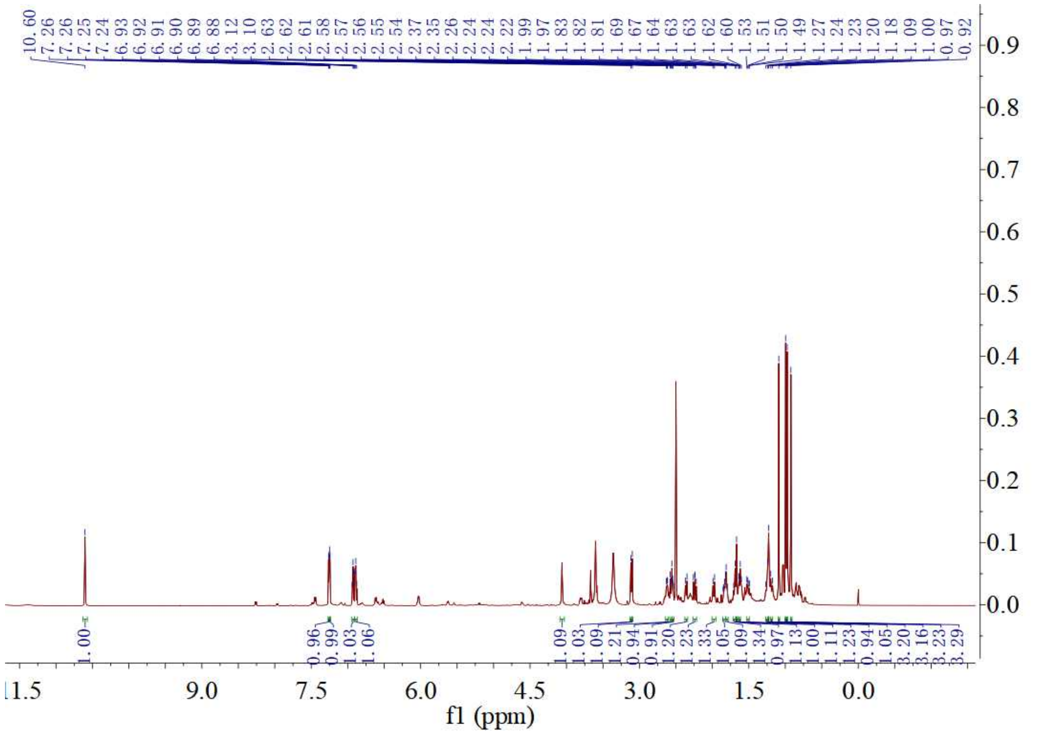

Figure S16. ^{1}H NMR spectrum of compound **4** in DMSO-d_6.

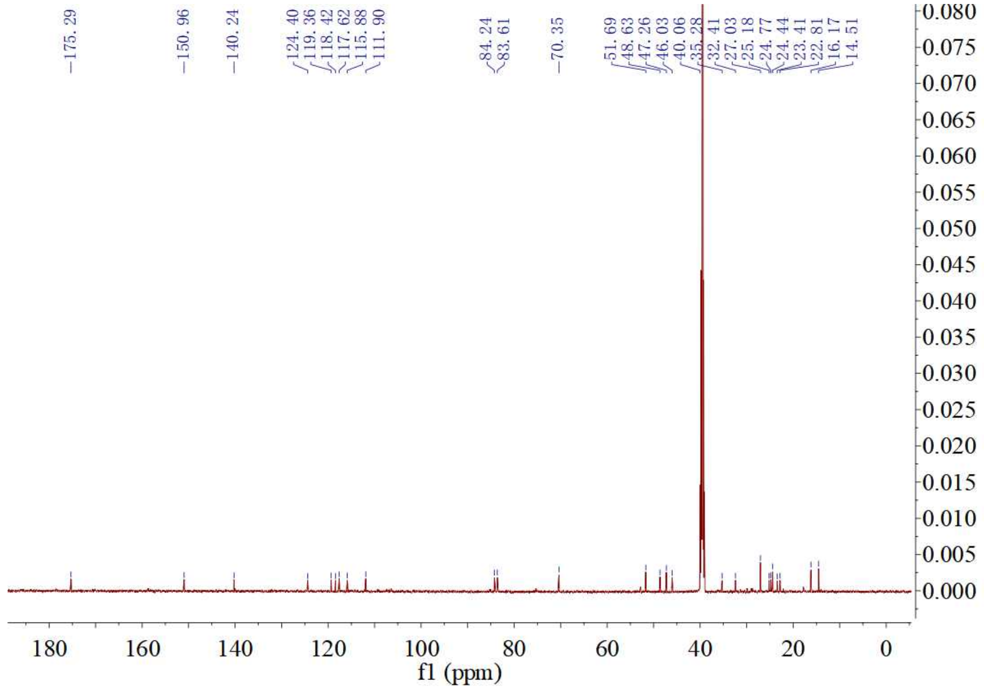

Figure S17. ^{13}C NMR spectrum of compound **4** in DMSO-d_6.

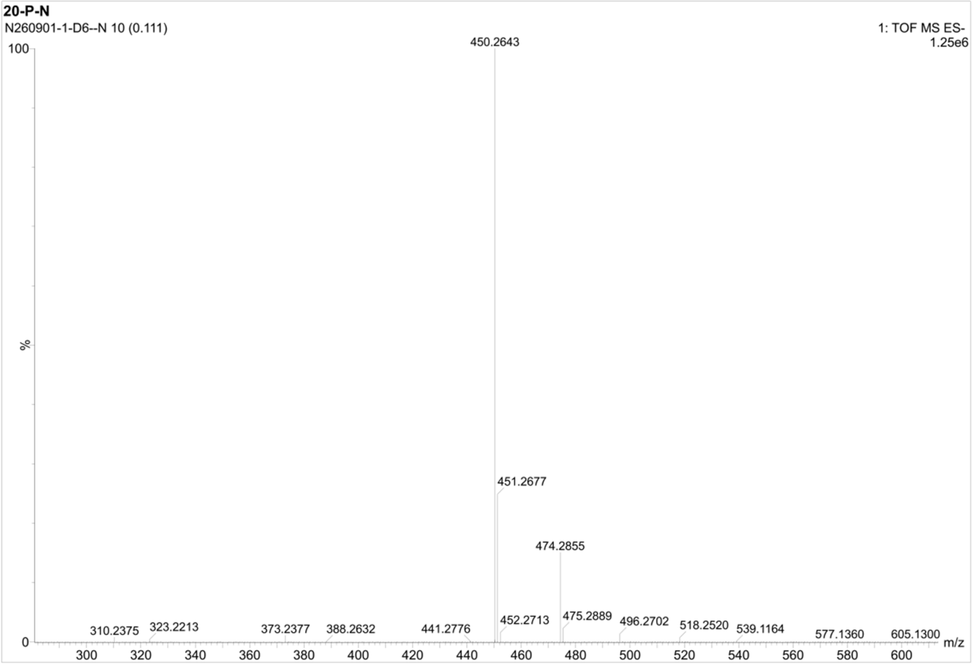

Figure S18. (–)-HRESIMS spectrum of compound **4.**

References

1. Mantle, P.G.; Weedon, C.M. Biosynthesis and transformation of tremorgenic indole-diterpenoids by *Penicillium paxilli* and *Acremonium lolii*. *Phytochemistry*, **1994**, *36*, 1209–1217.
2. Liang, Z.Y.; Shen, N.X.; Zhou, X.J.; Zheng, Y.Y.; Chen, M.; Wang, C.Y. Bioactive indole diterpenoids and polyketides from the marine-derived fungus *Penicillium javanicum*. *Chem. Nat. Comp*., **2020,** *56*, 379–382.
3. Liu, M.C.; Zhang C.J.; Liu D. Study on secondary metabolites of sponge-associated fungus *Penicillium janthinellum*. *Chin. Mar. Drugs* **2019,** *38,* 21-26.
4. Gallagher, R.T.; Fier, J.; Clardy, J. Paspalinine, a tremorgenic metabolite from *Claviceps paspalj* stevens et Hall. *Tetrahedron Lett.* **1980**, *21*, 235–238.
5. Fan, Y.Q.; Wang, Y.; Liu, P.P.; Fu, P.; Zhu, T.H.; Wang, W.; Zhu, W.M. Indole-diterpenoids with anti-H1N1 activity from the aciduric fungus *Penicillium camemberti* OUCMDZ-1492. *J. Nat. Prod*. **2013**, *76*, 1328‑1336.
